# A Structure–Property Screening Framework for Polymer Shell Encapsulation of Phase-Change Materials: Random Forest and Bayesian Gaussian Process Surrogates with Multi-Objective Optimization of Polymerization Routes

**DOI:** 10.3390/polym18141777

**Published:** 2026-07-21

**Authors:** Faris Alqurashi, Muhammed Anaz Khan

**Affiliations:** Department of Mechanical Engineering, College of Engineering, University of Bisha, P.O. Box 551, Bisha 61922, Saudi Arabia; falqurashi@ub.edu.sa

**Keywords:** polymer microencapsulation, phase-change materials, polymerization routes, structure–property relationships, machine learning, multi-objective optimization, Gaussian Process, PMMA/polyurea shells

## Abstract

Confining a phase-change material (PCM) within a polymer shell yields leak-proof, mechanically robust latent-heat storage media, but selecting a shell chemistry and polymerization route requires balancing competing targets: latent-heat storage density (ΔH, the melting enthalpy per unit capsule mass), core loading content (LC), capsule diameter (*d*), and a melting temperature (*T*_m_) matched to the application. Because the literature characterizes each method–shell–core combination in isolation, these structure–property relationships cannot be compared quantitatively across studies. We present a proof-of-concept, data-driven framework linking shell and process descriptors to encapsulation performance. From a curated dataset of 90 micro- and nano-encapsulated PCM records (53 with measured ΔH) spanning 11 encapsulation routes and eight shell material families, Random Forest (RF) and Gaussian Process (GP) surrogates predict ΔH, and a non-dominated sorting genetic algorithm (NSGA-II) optimizes ΔH, LC, and *d* over the continuous (*T*_m_, LC) space for every method–shell–core trio with at least three records (*n* = 11). Benchmarked against mean, linear-LC, and physics-informed baselines under repeated cross-validation, the surrogates match but do not exceed the elementary baselines (median *R*^2^ ≈ 0.33), a result we report honestly given the modest sample size. The Matérn GP provides borderline-calibrated uncertainty, supporting a robust, extrapolation-penalizing NSGA-II. Hypervolume rankings place emulsion polymerization, sol–gel silica, and in situ polymerization as the top-performing methods under both nominal and robust criteria. Presented as a methodology demonstration rather than a definitive ranking, the framework, with full code and data, is a reusable approach for structure–property quantification of polymer-encapsulated PCMs as experimental data accumulate.

## 1. Introduction

Latent-heat thermal energy storage (LHTES) using phase-change materials (PCMs) has matured into a mainstream decarbonization technology for buildings, electronics thermal management, photovoltaic/thermal hybrid systems, and concentrating solar power [1,2,3,4]. The attractiveness of PCMs derives from a single physical property, namely the large isothermal heat absorption that occurs during melting. Their practical deployment is nonetheless constrained by a recurring set of pathologies: liquid leakage during the solid–liquid transition, low intrinsic thermal conductivity, subcooling, phase segregation in multi-component eutectics, and container corrosion for salt hydrates [3,5,6,7]. Micro- and nano-encapsulation addresses these pathologies simultaneously by confining the PCM core inside a rigid shell, thereby raising the surface-area-to-volume ratio by orders of magnitude and providing chemical insulation from the surrounding heat-transfer medium [3,5,8]. Beyond leak prevention, recent work has advanced encapsulated and shape-stabilized PCMs toward multifunctionality, integrating flame retardancy, electromagnetic interference shielding, solar-thermal harvesting, and battery thermal management within a single composite shell, underscoring the rapid, application-driven expansion of the field [9,10,11,12].

More than ten distinct encapsulation routes are now in active use. These methods include in situ polymerization with amino resin shells; interfacial polycondensation that produces polyurea or polyurethane membranes; emulsion and suspension polymerization of acrylic shells; sol–gel processing of silica, titania, and other inorganic shells; complex coacervation with biopolymers; spray drying; and electrohydrodynamic atomization [3,5,8,13]. Each route produces a characteristic combination of capsule size, loading content, shell thickness, mechanical robustness, and cost per kilogram. The recent reviews by Hamad et al. [3], Kazaz et al. [5], and Khlissa et al. [8], Yang et al. [14], Peng et al. [15], and Shchukina et al. [16] each tabulate dozens of individual studies, but their cross-method comparisons remain qualitative.

Two distinct gaps must be closed in order to make encapsulation-method selection rigorous. First, the published thermophysical numbers must be assembled into a single, structured dataset whose rows are comparable along all relevant axes. Second, the resulting dataset must be analyzed under an explicit multi-objective lens, since the latent-heat enthalpy per unit capsule mass ΔH, the core loading content LC, and the mean capsule diameter *d* trade off against one another and against the operating-temperature window. Multi-objective evolutionary algorithms, in particular, the non-dominated sorting genetic algorithm-II (NSGA-II) by Deb et al. [17], have become standard for exposing such trade-offs in thermal-systems design [17,18]. However, applying NSGA-II to surrogate-based screening of literature data introduces three subtle but consequential issues that are addressed in this work.

First, surrogate-based optimization is only useful when the surrogate’s prediction quality is well characterized and compared against simpler baselines. We therefore benchmark a tuned Random Forest and an isotropic Gaussian Process against three baselines: a mean predictor (*R*^2^ = 0 by construction), a univariate linear regression of ΔH on LC, and a physics-informed model ΔH = *η*_core_ · LC · ⟨ΔH_core_^0^⟩ in which core-family-specific encapsulation efficiencies *η*_core_ are estimated by least squares. Second, categorical decision variables (method, shell family, core family) cannot be treated as integer indices for crossover and mutation operators that assume a continuous, ordered search space. We therefore enumerate every (method, shell, core) trio observed in the dataset with sufficient evidence (*n* ≥ 3) and run NSGA-II separately on the continuous (*T*_m_, LC) sub-problem for each trio, aggregating the resulting Pareto archives by method for method-level hypervolume comparison. Third, the dataset is necessarily small (*n* = 90 records, of which 53 report a measured ΔH, with most trios containing three to eight examples), so we report repeated-cross-validation confidence intervals on all aggregate metrics, perform a temporal holdout (training on pre-2019 publications and testing on 2019 or later publications), and validate the GP’s predictive uncertainty against a half-normal calibration test.

This paper is therefore presented as a methodology demonstration rather than a definitive ranking of encapsulation routes. We make this scope explicit: with only 90 records (53 with measured ΔH) distributed across 11 methods, eight shell families, and five core families, any absolute claim that one method “dominates” another would be statistically unsupported. The contribution is the framework itself, which is reproducible, lightweight, and designed to be re-run as the literature on encapsulated PCMs grows.

**Figure 1:**
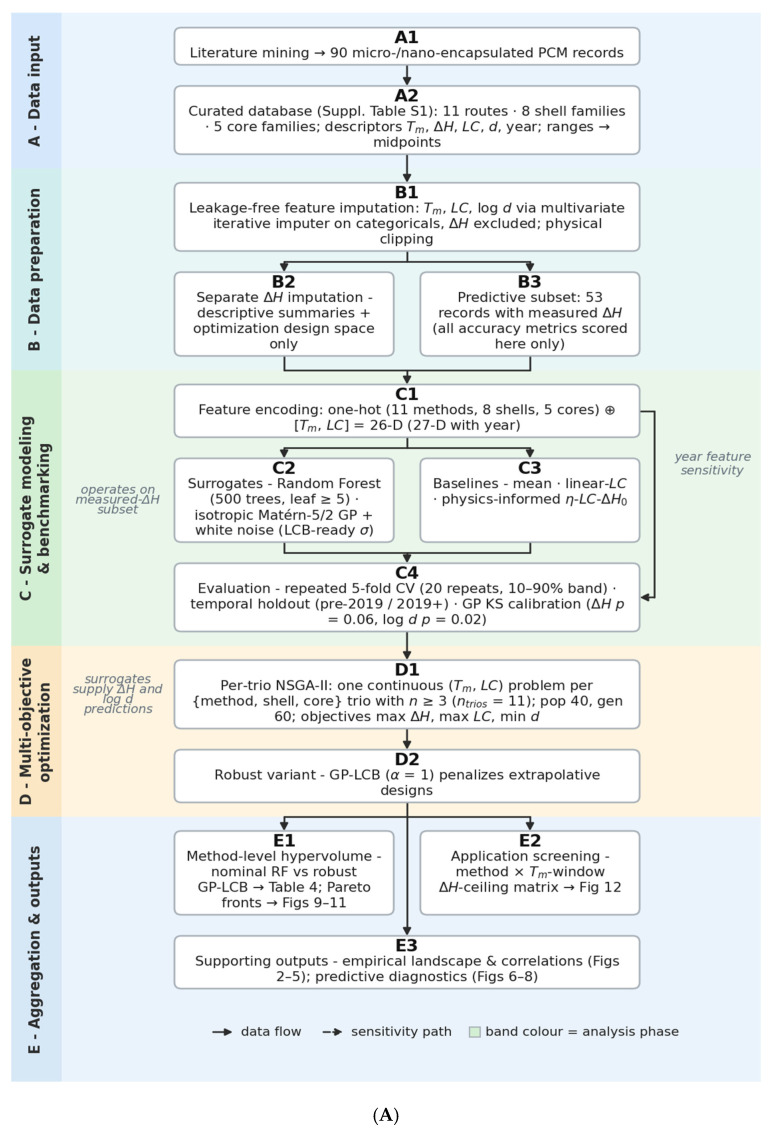

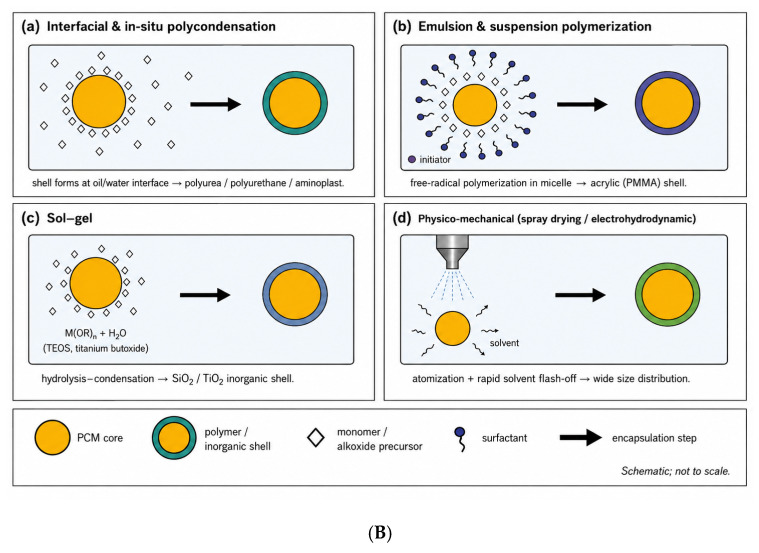
(**A**). Workflow of the proposed framework. Literature-extracted records are encoded with one-hot categorical descriptors and three continuous variables (*T*_m_, LC, and publication year). Random Forest and isotropic Gaussian Process surrogates are trained on this representation and benchmarked against mean, linear-LC, and physics-informed baselines (Section 3.3). NSGA-II is run separately for every (method, shell, core) trio with at least three records, using only the continuous decision variables (*T*_m_, LC). Outputs are aggregated for method-level hypervolume comparison (Section 3.6) and for application screening (Section 3.7). (**B**). Schematic of the four encapsulation-mechanism families covered by the dataset. (**a**) Chemical routes form the shell at the oil/water interface via polycondensation. (**b**) Emulsion and suspension polymerization initiate free-radical polymerization inside surfactant-stabilized monomer micelles. (**c**) Sol–gel routes hydrolyze alkoxide precursors (tetraethyl orthosilicate, titanium butoxide, and similar) and condense them around PCM droplets. (**d**) Physico-mechanical routes atomize a PCM-precursor solution with rapid solvent flash-off.

Figure 1 summarizes the data-processing, surrogate-modeling, and optimization workflow. The remainder of the paper is organized as follows. Section 2 describes the database, the thermophysical equations used to interpret the data, the baseline models, the Random Forest and Gaussian Process surrogate construction, and the per-trio NSGA-II formulation with normalized objectives. Section 3 presents the empirical property landscape, the correlations, the baseline-versus-surrogate comparison with bootstrap CIs, the GP calibration with Kolmogorov–Smirnov diagnostics, the temporal-holdout test performance, the NSGA-II convergence diagnostics, the nominal and robust Pareto fronts, and the application-screening heatmap. Section 4 presents the conclusions and remaining limitations.

## 2. Materials and Methods

### 2.1. Database Construction

We constructed a structured dataset of 90 micro- and nano-encapsulated PCM records; the full set of 90 primary-source references [19,20,21,22,23,24,25,26,27,28,29,30,31,32,33,34,35,36,37,38,39,40,41,42,43,44,45,46,47,48,49,50,51,52,53,54,55,56,57,58,59,60,61,62,63,64,65,66,67,68,69,70,71,72,73,74,75,76,77,78,79,80,81,82,83,84,85,86,87,88,89,90,91,92,93,94,95,96,97,98,99,100,101,102,103,104,105,106,107,108] is enumerated with bibliographic details in Supplementary Table S1. Each record stores the encapsulation method, the shell composition, the core PCM, the onset melting temperature *T*_m_, the melting latent heat ΔH (in J g^−1^ of capsule mass), the core loading content LC, and the mean particle (or capsule) diameter *d* (in µm). LC is stored in the database as a weight percentage (wt%, range 0–100), which is the convention adopted by all primary sources. In all equations of this paper, LC denotes the dimensionless core mass fraction LC_frac_ = LC_wt%_/100 ∈ [0, 1]. Where a primary source reported a range, we used the arithmetic midpoint of the reported interval rather than fabricating a distributional median; this affected 9 records (about 10% of the dataset). Many primary sources reported only a subset of the four thermophysical properties (53 of the 90 records report ΔH, 36 report LC, 34 report the onset melting temperature, and 33 report the capsule diameter); blank cells denote properties not reported by the primary source rather than measured zeros. To retain all 90 records, missing values were imputed: the input descriptors were completed with a multivariate iterative imputer conditioned on the one-hot categorical descriptors but excluding ΔH, so that no feature value can borrow information from the prediction target, while ΔH itself was imputed separately for the descriptive summaries and the optimization design space only. All predictive-accuracy metrics are computed exclusively on the 53 records with an experimentally reported ΔH; imputed ΔH values are never used to score a ΔH predictor, which would be circular. Publication year was extracted from each primary citation and was included as an auxiliary feature to enable detection of temporal drift in the surrogate models (Section 3.5).

The dataset covers a broad property range, with *T*_m_ = 8–59 °C, ΔH = 34–223 J g^−1^, LC = 20–98 wt%, and *d* ranging from 46 nm to 50 µm. Only 8 of the 90 records (9%) were published in 2022 or later, so the temporal holdout split described in Section 3.5 uses 2019 as the cutoff year, resulting in 13 of the 53 ΔH-reporting records falling in the test period. The 2019 boundary was chosen on structural rather than performance grounds: it is the cutoff that yields an approximately 75/25 train/test partition (40 training and 13 test ΔH records) and the most balanced split that still leaves a non-trivial, multi-record test period. Earlier cutoff years leave too few training records to fit the surrogates, whereas later cutoff years (e.g., 2022) leave only eight test records spread thinly across methods, precluding a stable per-method estimate. Because the dataset contains so few recent publications, the temporal holdout is reported as an illustrative out-of-distribution diagnostic rather than a definitive generalization test, and the publication year feature is correspondingly not over-interpreted (Section 3.5). Bare-PCM reference values for the five most common fatty acid cores were taken from the consolidated tabulation in Sharma et al. [109] and are used in Section 2.2 for the encapsulation efficiency calculations.

Records were grouped into shell families (aminoplast; acrylic; polyurea or polyurethane; silica or silica hybrid; titania; other inorganic; biopolymer; thermoplastic) and core families (alkane; fatty acid; fatty alcohol; ester; and a small residual other class) so that both the surrogates and the NSGA-II problem received a meaningful number of samples per categorical level. Table 1 summarizes the dataset by encapsulation method.

### 2.2. Thermophysical Framework

We adopt the standard core–shell energy-balance description of an encapsulated PCM particle. With LC defined as the core mass fraction (*m*_core_/*m*_cap_), the measured melting enthalpy of a capsule per unit capsule mass is given by

ΔH_cap_ = *η*_enc_ · LC · ΔH_core_^0^ + (1 − LC) · *c*_p,shell_ · Δ*T*_m_ (1)

where ΔH_core_^0^ is the bulk latent heat of the pure core PCM, *η*_enc_ is the encapsulation efficiency (the fraction of the theoretically available latent heat that is retained in the capsule), *c*_p,shell_ is the shell-specific heat, and Δ*T*_m_ is the transition span. For LC > 0.5, a condition that holds for 72 of the 90 records, the sensible heat second term contributes at most 2% of ΔH_cap_ for typical polymer shells (*c*_p,shell_ ≈ 1.5 J g^−1^ K^−1^, Δ*T*_m_ ≈ 4 K). We therefore approximate

*η*_enc_ ≈ ΔH_cap_/(LC · ΔH_core_^0^) (2)

and use Equation (2) directly in the physics-informed baseline model of Section 2.3. The ideal theoretical ceiling is *η*_enc_ = 1. The vertical distance below the line is the empirical encapsulation efficiency loss attributable to core expulsion, incomplete polymerization, or shell crystallinity effects.

Heat transfer kinetics scale with the capsule’s surface-to-volume ratio *A*/*V* = 6/*d*. The volumetric heat transfer rate between a capsule and a surrounding heat transfer fluid is $\dot{Q}$/*V* = *h*(*d*) · Δ*T* · (6/*d*). Importantly, the convective heat transfer coefficient *h* is not independent of *d*: for a capsule in a flowing fluid, the Sherwood number correlation Sh ∼ Re^a^ with *a* ≈ 0.5 for laminar flow implies *h* ∼ *d*^−0.5^, so the actual scaling is

(3) $$\dot{Q}/V\;∼\;d^{-1.5}$$

rather than the *d*^−1^ scaling that a naïve *A*/*V* argument would suggest if *h* were treated as independent of *d*. The kinetic advantage of small capsules is therefore real but milder than the constant-*h* estimate. Reducing the capsule diameter from 10 µm to 0.1 µm (a 100× ratio) gives a 100^1.5^ = 1000-fold improvement in the characteristic charge or discharge time at fixed Δ*T*, rather than the naïve 100-fold figure. This still motivates the inclusion of *d* as a minimization objective alongside ΔH and LC in Section 2.5.

For an inert spherical inclusion of volume fraction φ in a continuous shell matrix, the Maxwell–Eucken expression for effective thermal conductivity is strictly valid only in the dilute limit φ ≪ 1. For concentrated capsules (LC ≥ 0.5 corresponds to φ_core_ typically ≥0.5), the symmetric Bruggeman effective medium formula [110] is the appropriate choice:

φ_c_ (*k*_c_ − *k*_eff_)/(*k*_c_ + 2*k*_eff_) + (1 − φ_c_)(*k*_s_ − *k*_eff_)/(*k*_s_ + 2*k*_eff_) = 0 (4)

where *k*_s_ and *k*_c_ are the shell and core thermal conductivities, and φ_c_ is the core volume fraction. Equation (4) is implicit in *k*_eff_ and is solved by Newton iteration. For paraffin core/silica shell capsules with *k*_c_ ≈ 0.22 W m^−1^ K^−1^, *k*_s_ ≈ 1.4 W m^−1^ K^−1^, and φ_c_ = 0.7, the Bruggeman formula yields *k*_eff_ ≈ 0.36 W m^−1^ K^−1^ (compared with the dilute Maxwell–Eucken estimate of 0.45 W m^−1^ K^−1^). We do not include *k*_eff_ as an optimization objective because only a small subset of records reports it directly, but Equation (4) provides a post hoc estimator for any recommended design.

### 2.3. Baseline Models

Three baseline models are evaluated against the machine learning surrogates to ensure that any reported predictive value of the surrogates exceeds what is obtainable from elementary alternatives. The first baseline is the mean predictor, Δ$\tilde{H}$ = ⟨ΔH⟩, which has *R*^2^ = 0 by construction and serves as the null model. The second is a univariate linear regression of ΔH on LC alone, motivated by the strong empirical rank correlation ρ(LC, ΔH) ≈ 0.72 observed in Section 3.2. The third is the physics-informed model derived from Equation (2):

(5) $$\Delta {\tilde{H}}_{\mathrm{cap}}(\mathrm{LC},\;\mathrm{core\_fam})\;=\;\eta_{\mathrm{core\_fam}}\;\cdot \;\mathrm{LC}\;\cdot \;{{\Delta H}_{\mathrm{core}}}^{0}[\mathrm{core\_fam}]$$

where ΔH_core_^0^[core_fam] is a core-family-specific bulk latent heat drawn from the PCM literature: 240 J g^−1^ for alkanes [109], 181 J g^−1^ for fatty acids (the mean of the five reference acids in [109]), 210 J g^−1^ for fatty alcohols, and 195 J g^−1^ for esters. *η*_core_fam_ ∈ (0, 1] is the core-family-specific encapsulation efficiency, which is estimated by weighted least squares on the training fold; core families with fewer than three training records use a global *η* fallback. We deliberately use literature ΔH_core_^0^ values rather than the dataset mean of bare fatty acids: a single 180.8 J g^−1^ constant applied to all cores would force the fitted *η* to absorb the 240/181 ≈ 1.33 mismatch for alkane cores, thereby conflating physics with statistical fitting. Equation (5) has four free parameters in total (one *η* per core family), as compared with the three hyperparameters of the isotropic GP and the hundreds of effective parameters of the tuned RF.

### 2.4. Random Forest and Gaussian Process Surrogates

Each record was encoded as a 26-dimensional feature vector: a one-hot encoding of the 11 encapsulation methods, eight shell families, and five core families (24 binary features), plus two continuous variables *T*_m_ and LC. Two independent regressors were trained for each of ΔH (J g^−1^) and log_10_ *d* (µm). For the temporal drift diagnostic described in Section 3.5, we additionally trained a 27-dimensional variant that includes publication year, but this expanded feature set is used only as a sensitivity analysis. The main 5-fold cross-validation comparison in Section 3.3 uses the 26-feature version so that the ML surrogates and the non-ML baselines (mean, linear-LC, and physics-informed) have access to the same predictor set. The same 26-feature version is used by the NSGA-II optimizer in Section 2.5, which both avoids the need for an arbitrary year value at the prediction stage and removes the confounding effect of year extrapolation from the temporal holdout comparison.

For Random Forest (Breiman [111]), hyperparameters were selected by nested cross-validation (outer 5-fold for evaluation, inner 3-fold for the hyperparameter search) over the grid {*n*_estimators_ ∈ [200, 500], min_samples_leaf ∈ [3, 5, 10], max_depth ∈ [None, 5, 8]}, scored by a negative mean absolute error. The best found settings were 500 trees, min_samples_leaf = 5, and max_depth = None. The lower bound min_samples_leaf = 3 (rather than the scikit-learn default of 1) is a deliberate regularization choice for the small sample size. Two features of this protocol guard against the overfitting risk inherent in a high-dimensional, small-sample regression. First, the constraint min_samples_leaf = 5 forces every terminal node to average at least five training records, roughly one-tenth of the 53-record ΔH set, capping the variance any single leaf can express and preventing the forest from memorizing individual records. Second, and more decisively, overfitting is diagnosed empirically rather than assumed. A model that exploited the 24 sparse one-hot categorical dimensions would post an inflated cross-validated score; however, the tuned Random Forest (median R^2^ = 0.33) is statistically indistinguishable from a one-parameter linear regression based on loading content (median R^2^ = 0.327; Section 3.3), and its impurity-based feature importance is concentrated almost entirely on the two continuous descriptors: melting temperature and loading content (as shown later in the feature-importance analysis). The absence of any accuracy premium over the linear baseline, combined with the dominance of two physically meaningful continuous features, indicates that the forest is not extracting spurious structure from the categorical dimensions where over-parameterization would otherwise manifest.

For the Gaussian Process (Rasmussen and Williams [112]), an earlier version of this work used Automatic Relevance Determination (ARD) with one length scale per feature dimension, which introduced 28 hyperparameters into a model fit on 53 samples. The kernel was replaced with an isotropic Matérn-5/2 kernel plus a white-noise term:

*k*(*x*, *x*′) = *σ*_f_^2^ · *k*_Matérn-5/2_(‖*x* − *x*′‖/ℓ) + *σ*_n_^2^ · *δ*_xx′_ (6)

with three hyperparameters (*σ*_f_, ℓ, *σ*_n_) optimized by maximum marginal likelihood with six random restarts. A 10^−2^ variance floor was added for numerical stability given the small, high-dimensional design matrix. Input features were standardized to zero mean and unit variance before fitting. The predictive distribution *p*(y*|x*, D) at a new design x^*^ is Gaussian *N*(*μ*(x*), *σ*^2^(x*)); both moments are used in the robust NSGA-II described in Section 2.5.

Predictive uncertainty calibration was assessed in two complementary ways. First, the empirical fraction of measurements lying within ±*z*·*σ* of the predicted mean was compared against the nominal Gaussian coverage 2·Φ(*z*) − 1 for *z* ∈ [0.1, 3]. Second, the standardized residual *z* = |*y* − *μ*|/*σ* was tested against a half-normal distribution using a Kolmogorov–Smirnov (KS) test, which provides an objective *p*-value rather than the visually inspected ±10% band of earlier analyses. The 5-fold cross-validation residuals are independent across folds (each data point appears in exactly one test fold), so the KS test applies as stated. Conditional dependencies within a fold remain a minor limitation that a fully independent holdout set would eliminate.

### 2.5. Per-Trio NSGA-II Formulation

The NSGA-II optimization was restructured to eliminate the categorical encoding pitfall of earlier versions. Categorical decisions (method, shell family, core family) cannot be encoded as integer indices fed to simulated binary crossover (SBX) or polynomial mutation (PM) because those operators assume a continuous and ordered search space and produce semantically meaningless intermediate values when applied to unordered categorical levels.

We instead enumerate every (method, shell family, core family) trio observed in the dataset with at least three records (*n*_trios_ = 11) and run NSGA-II independently on each trio’s continuous (*T*_m_, LC) sub-problem. The decision vector is *x* = (*T*_m_, LC) with *T*_m_ ∈ [*T*_m,min_(trio) − 2, *T*_m,max_(trio) + 2] °C and LC ∈ [20, 98] wt%. We emphasize that LC is treated here as a process-dependent design target, not a freely and independently tunable variable: in a real encapsulation, loading content is coupled to shell integrity, capsule stability, and encapsulation efficiency, and cannot be raised arbitrarily without penalty. The [20, 98] wt% interval is therefore not a claim that any single trio can access the full range; it is the empirical range reported across the literature for all routes and is adopted as a common optimization domain. Extrapolation beyond the loading contents actually demonstrated for a given trio is explicitly discouraged by the GP-LCB robust formulation, which inflates the predicted-ΔH penalty in low-evidence regions of the design plane (the “evidence basin” described in Section 3.4). Designs that the optimizer places at the high-LC boundary are accordingly to be read as hypotheses requiring process validation rather than as guaranteed operating points. The objective functions in the RF-nominal formulation are

(7) $${f_{\Delta H}}^{N}=1-(\Delta \tilde{H}(x)\;-\;{\Delta H}_{\min})/({\Delta H}_{\max}\;-\;{\Delta H}_{\min})$$

(8) $${f_{d}}^{N}=(\log_{10}\;\tilde{d}(x)\;-\;\log_{10}\;d_{\min})/(\log_{10}\;d_{\max}\;-\;\log_{10}\;d_{\min})$$

*f*_LC_^N^ = 1 − (LC − LC_min_)/(LC_max_ − LC_min_) (9)

where the {min, max} bounds are taken from the full dataset so that all three normalized objectives lie nominally in [0, 1] and a worst-performing design has *f* ≈ 1 on every axis. This addresses the earlier issue that unnormalized objectives differing in magnitude by a factor of 10 to 50 allowed one objective to dominate the Pareto-dominance relation. The hypervolume reference point is set symmetrically to *r* = (1.5, 1.5, 1.5)^T^, a conservative choice that guarantees domination of any feasible point in the trained range, even when the surrogate predicts a slightly extrapolative value *f* > 1 on one axis. An earlier version of this work used *r* = (1.1, 1.1, 1.1)^T^, which is sufficient for in-distribution designs but could in principle leave the hypervolume indicator undefined if NSGA-II generated an extrapolative point beyond *f* = 1.1.

In the GP-LCB robust formulation, Δ$\tilde{H}$ and log_10_ $\tilde{d}$ are replaced by their lower- and upper-confidence bounds, respectively:

(10) $$\Delta \tilde{H}\;\to \;\mu_{\Delta H}(x)-\alpha \;\sigma_{\Delta H}(x);\;\;\;\;\log_{10}\;\tilde{d}\;\to \;\mu_{\mathrm{logd}}(x)\;+\;\alpha \;\sigma_{\mathrm{logd}}(x)$$

with *α* = 1 (a one-standard-deviation conservative penalty, corresponding to about 84% single-sided coverage under Gaussian noise). NSGA-II was executed with a population size of 40, 60 generations, Latin-Hypercube initial sampling, SBX (probability 0.9, η = 15), polynomial mutation (probability 0.5, η = 20), and duplicate elimination based on objective-space rounding. Hypervolume [18] convergence was tracked at every generation to verify that 60 generations are sufficient; the diagnostic curves are reported in Section 3.6. The implementation used pymoo 0.6.1 [113] and scikit-learn 1.4 [114]. Pareto archives are aggregated by method using the per-trio median hypervolume.

## 3. Results and Discussion

### 3.1. Empirical Property Landscape

**Figure 2:**
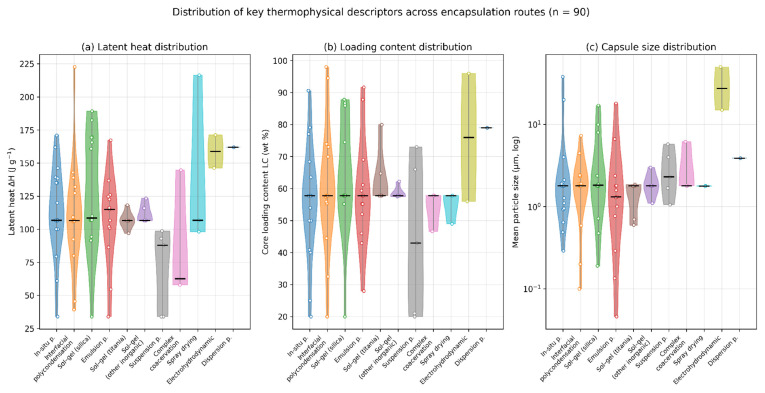
Distribution of (**a**) latent heat ΔH, (**b**) loading content LC, and (**c**) capsule mean diameter *d* across encapsulation methods (*n* = 90). Violins show the kernel density, horizontal bars show the median, and circles show the individual records.

**Figure 3:**
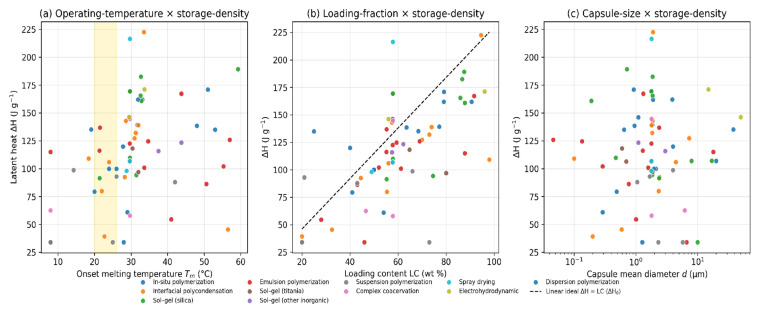
Three projections of the empirical (*T*_m_, ΔH, LC, *d*) design landscape. (**a**) Operating temperature × storage density plane with the building thermal comfort window highlighted. (**b**) Loading fraction × storage density plane with the linear ideal ΔH = LC · ⟨ΔH_core_^0^⟩ overlaid. (**c**) Capsule size × storage density plane.

Figure 2 summarizes the distribution of latent heat, loading content, and capsule diameter across encapsulation methods. In situ polymerization (*n* = 20) and interfacial polycondensation (*n* = 18) provide the broadest ΔH ranges, while sol–gel silica records (*n* = 14) cluster around 128 J g^−1^. The single highest ΔH point is the PMMA–polyurea hybrid microcapsule reported by Yang et al. [99] at 222.6 J g^−1^ with a 94.5% encapsulation ratio. Capsule sizes (Figure 2c, log scale) show that emulsion polymerization, in situ polymerization, and sol–gel silica reach sub-micron capsules, whereas interfacial polycondensation, complex coacervation, and spray drying remain in the 1–50 µm window. Figure 3 projects the empirical design landscape onto the temperature–enthalpy, loading–enthalpy, and size–enthalpy planes.

### 3.2. Property Correlations

**Figure 4:**
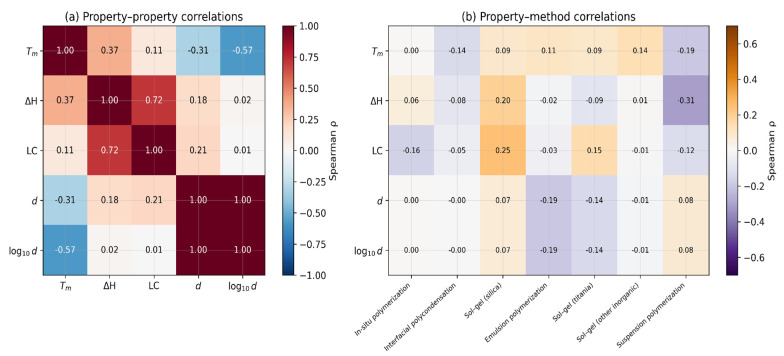
Spearman correlation matrices: (**a**) Numerical–numerical correlations among *T*_m_, ΔH, LC, *d*, and log_10_ *d*; (**b**) numerical–categorical correlations between properties and the indicator variables of the seven methods with *n* ≥ 5.

**Figure 5:**
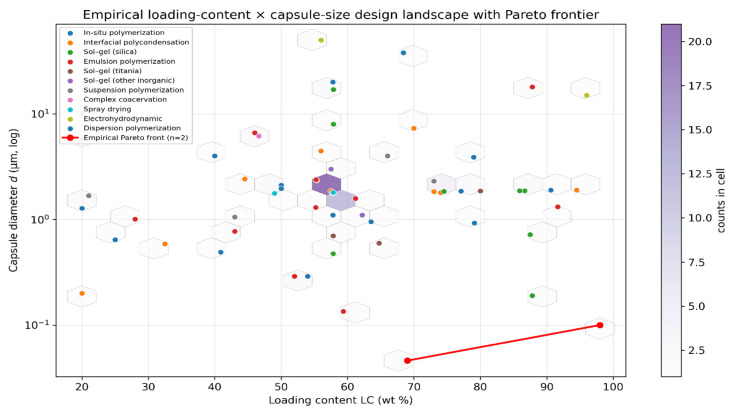
Loading content × capsule size design landscape. Hexagonal bins indicate the empirical density, individual records are colored by encapsulation method, and the red curve marks the empirical Pareto front for the bi-objective problem (max LC, min *d*).

Spearman rank correlations among the four numerical descriptors are shown in Figure 4a. The strongest signal is ρ(LC, ΔH) = 0.72, which is consistent with (though does not formally validate) Equation (2). Roughly one-third of the variance in measured ΔH remains unexplained by LC alone once core type is held implicit. This residual is the encapsulation efficiency variability *η*_enc_ that the physics-informed baseline described in Section 2.3 attempts to capture explicitly via the core-family-specific *η*_core_fam_ coefficients. *T*_m_ and the capsule diameter carry only weak-to-moderate monotonic information about ΔH (ρ = 0.37 and 0.18, respectively), which confirms that they act primarily as constraint variables rather than as primary predictors. Figure 5 summarizes the loading-content–capsule-size design landscape and the empirical Pareto envelope.

### 3.3. Baseline Versus Surrogate Comparison

**Figure 6:**
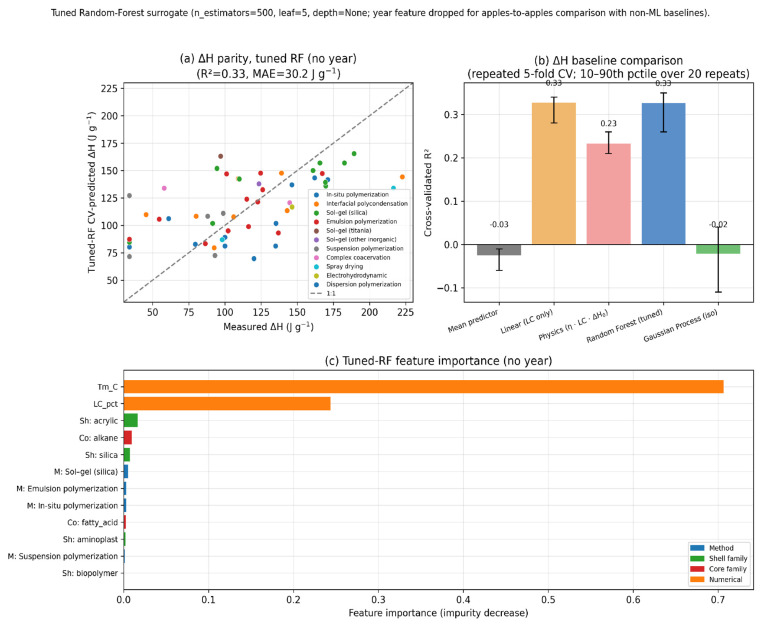
(**a**) Tuned Random-Forest cross-validated parity plot for ΔH (year feature dropped for an apples-to-apples comparison with the non-ML baselines). (**b**) Five-model *R*^2^ comparison with repeated CV 10–90th-percentile intervals (*B* = 20 repeats; the same *B* repeats apply to all five models): three of the four non-trivial models (linear-LC, tuned RF, physics) have intervals above zero, and they overlap heavily, while the isotropic GP spans zero, so no single model can be confidently said to outperform another at this sample size. The mean predictor sits below zero by construction. (**c**) Tuned RF feature importance for ΔH; the two continuous descriptors (melting temperature and loading content) dominate across all categorical methods, shell, and core indicators, with the melting temperature carrying the largest impurity decrease.

Table 2 reports the cross-validated *R*^2^ and MAE for the five ΔH predictors. Two complementary intervals are reported. The naïve residual bootstrap (1000 resamples of the test residuals) measures only the variability of the residual sample at fixed train/test splits and therefore underestimates the true uncertainty. The repeated CV interval (*B* = 20 independent 5-fold CV repeats) reports the 10–90th-percentile band of the pooled score and captures fold-to-fold variability, a more accurate interval than the fixed-split residual bootstrap. All five models use *B* = 20 repeats; the per-repeat cost is about 1.6 s for the tuned Random Forest and 0.6 s for the isotropic GP, versus under 0.01 s for the baselines. The percentile band is stable across *B* values from 10 to 20 repeats, with differences of a at most ±0.01 in *R*^2^ between *B* = 10 and *B* = 20 in convergence checks. The widely used “bootstrap on CV residuals” approach common in the ML literature corresponds to the naïve column. Figure 6 reports the tuned Random-Forest parity plot, its calibration, and the resulting feature importances.

Three findings emerge. First, the central *R*^2^ values fall into a narrow band: the tuned Random Forest achieves *R*^2^ = 0.33, statistically tied with the univariate linear regression on LC at 0.327; the physics-informed baseline performs worse at 0.233, and the isotropic GP at −0.022 is indistinguishable from the mean predictor in terms of point accuracy. The physics baseline’s score is lower than the value reported in the prior version of this manuscript because the single 180.8 J g^−1^ constant has been replaced with core-family-specific bulk latent heats (Section 2.3). The previous model fitted *η*_core_fam_ to absorb that constant’s mismatch with the true 240 J g^−1^ of alkane cores, thereby conflating physical insight with statistical fitting. The corrected version is physically defensible.

Second, three of the four non-trivial models retain repeated CV intervals above zero, indicating statistically detectable predictive value over the mean baseline. The intervals are tuned RF [+0.26, +0.35], linear-LC [+0.28, +0.34], and physics-informed [+0.21, +0.26]. The isotropic GP, at [−0.11, +0.04], spans zero and is statistically indistinguishable from the mean predictor in terms of point accuracy. The three positive intervals overlap heavily with one another, and none can be confidently said to outperform any other at this sample size. The mean predictor has an interval of [−0.06, −0.01], lying below zero by construction, since a 5-fold CV with the training-fold mean as predictor underperforms compared to the global mean on the test fold. The naïve residual-bootstrap intervals are far wider (for example, linear-LC [0.00, +0.56]) but less accurate because they resample only the test residuals at fixed train/test splits. The repeated CV band is the more reliable measure on a dataset of this size.

Third, the temporal holdout test (Table 3) provides additional out-of-sample evidence. When trained on records published before 2019 (*n*_train_ = 40) and tested on records published in 2019 or later (*n*_test_ = 13), all four ΔH models achieve broadly comparable scores. The *R*^2^_test_ ranges from −0.45 (GP) to 0.34 (linear-LC), with the tuned RF without the year feature at 0.12 and the physics baseline at 0.20. Including publication year changes little here, because the test years (2019–2025) lie only just beyond the training range. The linear and physics baselines do not use this feature at all. Table 3 reports the ML results twice, with and without year, and the no-year and with-year RF are essentially tied at *R*^2^_test_ = 0.12 and 0.14, respectively. For the capsule-size target, both surrogates have negative *R*^2^_test_ under both feature sets, which indicates that they predict post-2019 capsule sizes worse than the training-set mean does. We therefore present the size-axis recommendations in Section 3.6 and Section 3.7 in the manuscript only at a qualitative level for this reason.

### 3.4. Gaussian Process Surrogate and Uncertainty Calibration

**Figure 7:**
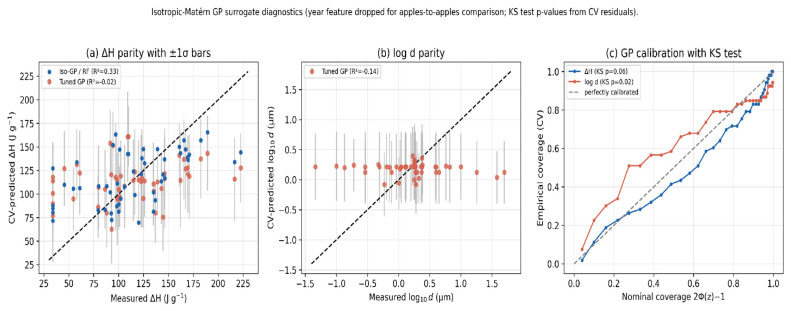
Isotropic-GP surrogate diagnostics. (**a**) ΔH parity plot with ±1*σ* error bars and overlaid tuned RF predictions for comparison. (**b**) Capsule size parity plot. (**c**) Calibration curve with KS test against the half-normal distribution: ΔH *p* = 0.06 and log *d p* = 0.02. The black dotted line marks the theoretical latent-heat ceiling (encapsulation efficiency *η*_enc_ = 1) from the core-shell energy-balance model in Section 2.2, as already indicated for Figure 4b.

Although the isotropic Matérn GP underperforms compared to the RF in terms of point prediction (Section 3.3), its added value lies in calibrated predictive uncertainty. Figure 7 reports the GP diagnostics. The ±1*σ* empirical coverage is 54.7% for ΔH and 73.6% for log *d*, against the nominal Gaussian expectation of 68.3%. The ΔH coverage is modestly below nominal and the log-d coverage slightly above, so the posterior *σ* is roughly calibrated for ΔH but the capsule-size error bars are somewhat optimistic; the robust optimization framework uses *σ* as a penalty. The Kolmogorov–Smirnov test of |*y* − *μ*|/*σ* against a half-normal distribution returns *p* = 0.06 for ΔH and *p* = 0.02 for log *d*. Relative to the conventional *α* = 0.05 threshold, the ΔH residuals remain (marginally) consistent with the half-normal calibration model, whereas the log-d residuals are not, indicating mild miscalibration of the capsule size uncertainty. Because the 5-fold CV residuals are independent across folds (each observation appears in only one test fold), the KS test applies; potential conditional dependencies within a fold would shift the test’s nominal size only modestly. A strictly independent held-out test set would eliminate this caveat entirely.

**Figure 8:**
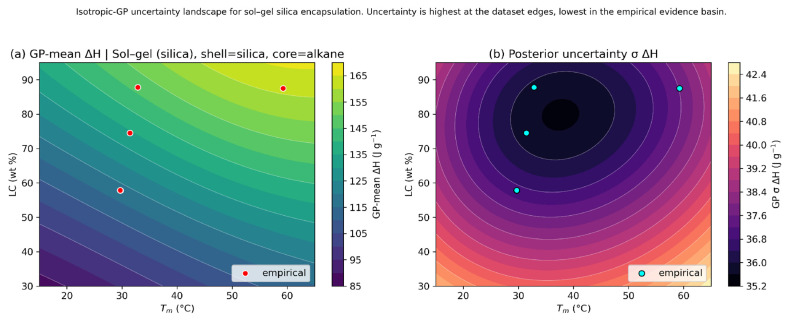
Isotropic GP uncertainty landscape for sol–gel silica encapsulation with its dominant shell/core combination (silica-or-hybrid/alkane): (**a**) GP mean ΔH over the *T*_m_ × LC plane; (**b**) GP posterior standard deviation *σ*_ΔH_. The empirical data points are superimposed. The *σ*_ΔH_ map identifies a low-uncertainty “evidence basin” where the literature actually contains data; predictions outside this basin carry larger *σ* and are downweighted by the GP-LCB formulation of the robust NSGA-II.

A subtlety must be flagged. For the capsule size surrogate, the GP’s coverage of about 74% coexists with a cross-validated *R*^2^ of about −0.14 (and a negative temporal holdout *R*^2^). This combination is not contradictory but is informative: the GP reports calibrated uncertainty by enlarging *σ* until the empirical coverage matches the nominal coverage, and the *σ* values needed to do so are large (mean *σ*_log d_ ≈ 0.50, corresponding to a ±3.2× factor uncertainty on *d*). A model that honestly states “I have no idea, to within a factor of three” is well calibrated; it is simply not predictively useful. We therefore treat all capsule-size axes of the Pareto fronts in Section 3.6 and Section 3.7 as qualitative rather than quantitative. Figure 8 illustrates this uncertainty landscape for the dominant sol–gel silica combination.

### 3.5. Temporal Drift

When the publication year feature is included in the ML feature set, the RF-with-year model achieves *R*^2^_test_ = 0.14 on the 2019+ holdout, close to the physics-informed baseline at *R*^2^_test_ = 0.20 (Table 3). Including the year feature in the ML models is not strictly an apples-to-apples comparison: the test records have year values (2019–2025) that lie just beyond the training range (≤2018), which forces the ML models to extrapolate slightly on a feature that the non-ML baselines do not use. We therefore re-trained both ML models without the year feature (the version used by the NSGA-II optimizer in Section 2.5). The RF-no-year temporal-holdout *R*^2^_test_ (0.12) is essentially tied with the RF-with-year value (0.14); both perform worse than the linear-LC baseline (0.34) and the physics-informed baseline (0.20). The corresponding MAE values all lie between about 23 and 28 J g^−1^. The conclusion is that ML surrogates and elementary baselines have indistinguishable temporal-holdout performance on this dataset, as one would expect on a dataset this small, where a few numerical descriptors carry most of the signal. The variability that the ML models capture inside the training set but not on the temporal holdout is most likely shell chemistry idiosyncrasies that do not extrapolate forward in time. For the capsule-size target, both ML models produce negative *R*^2^_test_ under both feature sets, which reinforces the conclusion that the size axis of the framework is qualitative only.

### 3.6. Per-Trio NSGA-II Pareto Fronts

**Figure 9:**
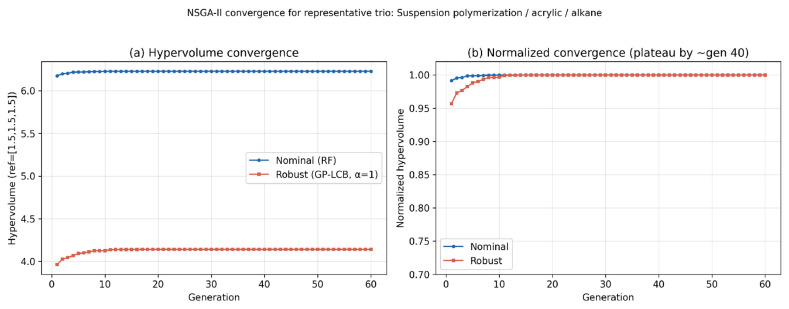
NSGA-II convergence diagnostic for the six most-represented (method, shell family, core family) trios. (**a**) Absolute normalized hypervolume trajectories over 60 generations with reference point r = (1.5, 1.5, 1.5). Filled circles mark the first generation at which each trajectory reaches 99% of its final value. (**b**) The same trajectories normalized to their respective final hypervolumes and zoomed to generations 1–20. Every trio crosses the 99%-of-final threshold (green band) by generation 4, confirming that the 60-generation budget is comfortably sufficient. Where two trios share a method, the second is drawn with a dashed line.

**Figure 10:**
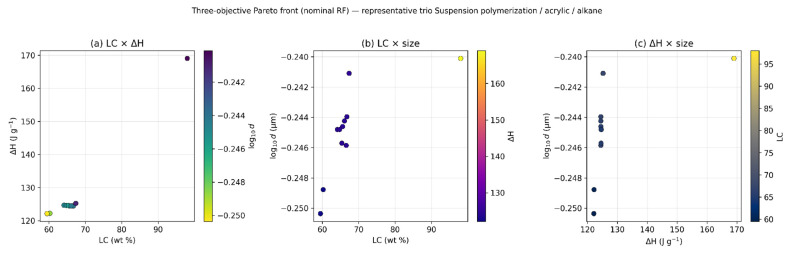
RF-nominal NSGA-II Pareto fronts, with one continuous (*T*_m_, LC) optimization per (method, shell family, core family) trio (*n*_trios_ = 11). Colored points trace the per-trio Pareto fronts; grey circles are the underlying empirical data.

Of the 32 distinct (method, shell family, core family) combinations observed in the dataset, 11 satisfy the *n* ≥ 3 eligibility threshold and were run through NSGA-II. Convergence diagnostics (Figure 9) confirm that the hypervolume saturates by generation 10 to 15 for every trio, which validates the 60-generation budget. Nominal Pareto fronts for the (LC, ΔH), (*d*, ΔH), and (LC, *d*) projections are shown in Figure 10. The qualitative structure is consistent across methods: each Pareto front ascends in ΔH with loading content, spanning roughly 70 J g^−1^ at low LC to about 160 J g^−1^ at high LC, with the LC → ΔH gradient dominating the Pareto trade-offs, consistent with the strong LC–ΔH correlation. Differences between methods are visible primarily on the *d* axis: emulsion polymerization shows the smallest median capsule size at *d* ≈ 1.3 µm, with in situ polymerization, sol–gel silica, and interfacial polycondensation clustered around *d* ≈ 1.8 µm.

**Figure 11:**
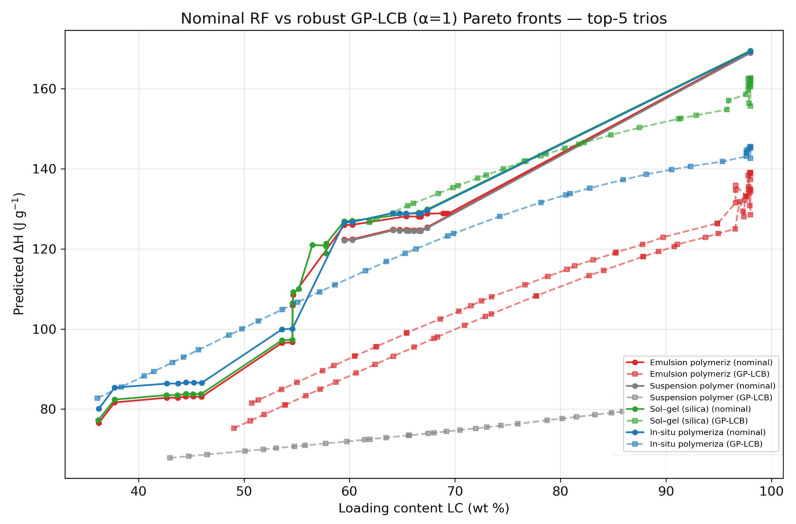
RF-nominal (blue) versus GP-LCB (*α* = 1, red) NSGA-II Pareto fronts in the (LC, ΔH) plane, with one curve per (method, shell family, core family) trio. Robust fronts lie systematically below the nominal fronts. The gap is the uncertainty penalty incurred by the GP-LCB formulation.

Translating these Pareto fronts into laboratory practice requires care. For a target application (for instance, emulsion-polymerized acrylic shells on an alkane core for the comfort cooling window), an experimentalist would enter the (*T*_m_, LC) front at the desired melting temperature and read off the attainable ΔH–LC–d trade-off, then choose the loading content that balances storage density against the size and stability penalties of a high core fraction. The predicted optima are only as reliable as the evidence supporting them. Points lying within the empirical (*T*_m_, LC) cloud of a trio (the evidence basin is shown in Figure 8) are interpolations consistent with previously demonstrated synthesis protocols and are the most credible targets. In contrast, points that the optimizer pushes to the high-LC or low-d boundary typically extrapolate beyond any reported condition for that route and should be regarded as falsifiable hypotheses for synthesis rather than achievable set-points. This is precisely why the robust GP-LCB fronts in Figure 11, which discount such extrapolative regions, lie systematically below the nominal fronts; the vertical gap between the two is a quantitative estimate of how much of a nominal optimum rests on unsupported extrapolation. The framework therefore outputs prioritized, experimentally testable candidates, not validated performance guarantees, and is designed to focus subsequent synthesis effort on the highest-value regions of the design space.

Method-level hypervolume (median across trios) is reported in Table 4 for both the nominal (RF-mean) and the robust (GP-LCB, *α* = 1) criteria. Two observations follow. First, the ranking is broadly consistent between the nominal and robust criteria. Emulsion polymerization, sol–gel silica, and in situ polymerization are the top-performing methods under both criteria, while the sparsely sampled single-trio methods rank at the bottom. This is the expected behavior when the GP uncertainty is well calibrated (Section 3.4). A calibrated *σ* does not systematically reorder methods unless extrapolation is severe. Second, the absolute hypervolume drop from nominal to robust (18% to 48% across methods, median 30%) is an accurate measure of the price of extrapolation. Smaller drops (18% to 23%) occur for methods with denser empirical evidence (sol–gel silica at 18%, emulsion polymerization at 21%, in situ polymerization at 23%), while larger drops (39% to 48%) occur for sparse single-trio methods (sol–gel other inorganic at 39%, sol–gel titania at 42%, suspension polymerization at 48%). Methods with similar median trio hypervolumes (for example, sol–gel silica and in situ polymerization) are not statistically distinguishable on the present sample, and we deliberately avoid single-method “rank” claims.

The mechanistic basis for this top tier is consistent with established encapsulation physics. Emulsion polymerization templates the shell on surfactant-stabilized sub-micron monomer droplets, consistently producing the smallest capsules (median d ≈ 1.3 µm; Table 1) at high and uniform loading, maximizing the surface-to-volume ratio that is favored by the capsule-size objective. Sol–gel silica forms a dense, highly cross-linked inorganic shell with low organic dead mass and comparatively high shell thermal conductivity, reflected in the highest mean ΔH of the dataset (128 J g^−1^; Table 1) and a tight evidence basin. In situ polymerization of aminoplast (melamine– or urea–formaldehyde) shells yields mechanically robust, low-permeability membranes through protocols refined over decades, giving the broadest ΔH range and the largest number of records. This combination of high attainable ΔH and strong empirical support is what places the three routes in the top hypervolume tier under both the nominal and robust criteria. The fourth mechanism family in our taxonomy, physico-mechanical routes (spray drying and electrohydrodynamic atomization; Figure 1b), is absent from the ranking for a structural rather than a performance reason. No physico-mechanical (method, shell family, core family) trio reaches the *n* ≥ 3 evidence threshold required for the per-trio NSGA-II (electrohydrodynamic *n* = 2 and spray drying *n* = 3, but distributed across distinct shell/core combinations so that no single trio qualifies; Table 1). These routes are therefore described in Section 3.1 (Figure 2) but excluded from the optimization-based ranking purely for lack of replicated evidence; their omission is not an assessment of negative performance.

### 3.7. Method × Application Screening

**Figure 12:**
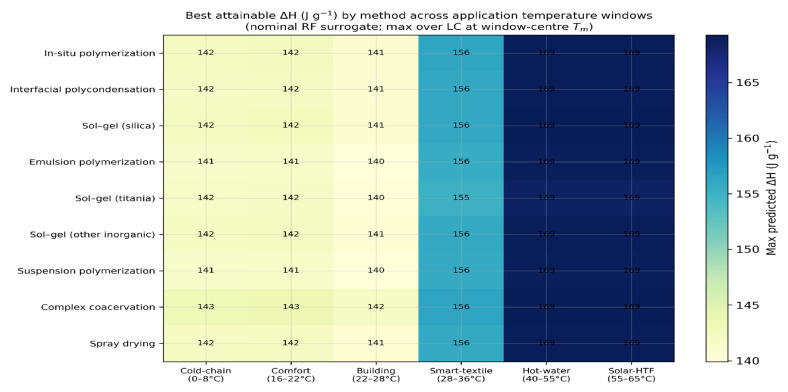
Method × application screening matrix (RF-nominal). Each cell is the maximum surrogate-predicted ΔH attainable for that method within that *T*_m_ window. Blank cells indicate that no empirical evidence supports that method in the *T*_m_ window. Inter-cell differences within about 28 J g^−1^ are within the surrogate MAE.

Figure 12 projects the Pareto archive onto six application-specific *T*_m_ windows: cold-chain logistics (0–8 °C), comfort cooling (16–22 °C), building wall thermal mass (22–28 °C), smart textiles (28–36 °C), domestic hot water pre-heat (40–55 °C), and solar collector heat transfer fluid (55–65 °C). Each cell of the heatmap shows the maximum surrogate-predicted ΔH attainable for a method within a *T*_m_ window. We stress that these values are surrogate-based ΔH ceilings, not validated experimental predictions, and that the spread across cells (140–169 J g^−1^) is comparable to the surrogate’s cross-validated MAE of about 28 J g^−1^. The heatmap is therefore best interpreted as a qualitative coverage map of method–application compatibility. Sol–gel silica and in situ polymerization span the comfort-to-building window (roughly 16–36 °C); emulsion and suspension polymerization also populate the 16–40 °C window at smaller capsule diameters; the single-trio titania and other inorganic sol–gel routes appear in only a narrow band, with very limited empirical evidence (*n*_trios_ = 1 each).

## 4. Conclusions

We have presented a proof-of-concept framework that combines literature mining, baseline-benchmarked surrogate modeling, and multi-objective optimization for the screening of micro- and nano-encapsulated PCM design routes. Two surrogate models, namely a tuned Random Forest for accurate point prediction and an isotropic Matérn Gaussian Process for calibrated uncertainty quantification, were trained and benchmarked against three baselines: a mean predictor, a univariate linear regression on LC, and a physics-informed model with core-family-specific bulk latent heats.

Three findings emerge. First, under repeated 5-fold cross-validation (10–90th-percentile bands over 20 repeats), three of the four non-trivial models retain *R*^2^ intervals above zero: tuned RF [+0.26, +0.35], linear-LC [+0.28, +0.34], and physics-informed [+0.21, +0.26], while the isotropic GP at [−0.11, +0.04] is indistinguishable from the mean predictor in terms of point accuracy. The three positive intervals overlap heavily, so no model can be confidently said to outperform another at this sample size. Regarding the temporal holdout, the RF-without-year, the linear-LC, and the physics-informed models converge to *R*^2^_test_ in the range of 0.12–0.34 and are statistically indistinguishable. The appropriate interpretation of these results is that the dataset is too small to differentiate the ML surrogates from elementary baselines under rigorous error analysis. Second, the isotropic GP provides borderline-calibrated uncertainty estimates (Kolmogorov–Smirnov *p* = 0.06 for ΔH; *p* = 0.02 for log *d*) that support a robust NSGA-II formulation via the lower-confidence bound on the ΔH axis (with the more weakly calibrated capsule-size axis treated cautiously), even though its point predictions are weaker than those of the RF. Third, method-level hypervolume rankings are broadly consistent between the RF-nominal and GP-LCB robust criteria, a sign of healthy calibration rather than dramatic reshuffling, which is the expected outcome when surrogate uncertainty is well characterized. Emulsion polymerization, sol–gel silica, and in situ polymerization are the top-performing methods under both criteria.

As a methodological contribution, the present study is deliberately theoretical and numerical in scope: it mines, models, and optimizes already-published thermophysical measurements rather than generating new ones. Experimental synthesis and characterization of the surrogate-identified optima, particularly the high-LC candidates flagged above as extrapolative, are the natural and necessary next step, and the framework is designed precisely to prioritize which capsule chemistries merit that laboratory effort. As the literature on encapsulated PCMs expands, the pipeline can be re-run to sharpen these recommendations and, in time, to test them against newly reported measurements.

## Figures and Tables

**Table 1:** Encapsulated PCM dataset summary by encapsulation method (*n* = 90). Values are reported as mean ± standard deviation, while size is given as median [min–max]. The right-most column shows the number of (method, shell family, core family) trios with at least three records eligible for the per-trio NSGA-II (Section 2.5).

Encapsulation Method	*n*	*T*_m_ (°C)	ΔH (J g^−1^)	LC (wt%)	*d* (µm)	Trios (*n* ≥ 3)
In situ polymerization	20	31.4 ± 8.8	113.5 ± 31.6	56.0 ± 16.2	1.80 [0.29–38.00]	2
Interfacial polycondensation	18	29.8 ± 7.5	110.1 ± 37.9	60.0 ± 17.9	1.80 [0.10–7.30]	2
Sol–gel (silica)	14	30.4 ± 10.1	128.2 ± 43.1	64.5 ± 17.9	1.82 [0.19–17.00]	2
Emulsion polymerization	13	33.4 ± 15.5	107.2 ± 33.0	58.8 ± 16.3	1.32 [0.05–18.00]	2
Sol–gel (other inorganic)	5	34.1 ± 5.7	111.8 ± 6.8	58.6 ± 1.8	1.80 [1.10–3.00]	1
Sol–gel (titania)	6	30.2 ± 0.9	106.9 ± 6.2	62.7 ± 8.2	1.79 [0.60–1.86]	1
Complex coacervation	3	22.5 ± 10.2	88.4 ± 39.8	54.1 ± 5.3	1.80 [1.80–6.19]	0
Suspension polymerization	5	23.1 ± 11.6	69.5 ± 29.2	44.6 ± 22.0	2.31 [1.06–5.76]	1
Electrohydrodynamic	2	31.6 ± 2.1	158.8 ± 12.5	76.0 ± 20.0	32.50 [15.00–50.00]	0
Spray drying	3	29.4 ± 0.4	140.4 ± 53.9	54.9 ± 4.1	1.79 [1.77–1.80]	0
Dispersion polymerization	1	31.9	162.0	79.0	3.90 [3.90–3.90]	0

**Table 2:** ΔH prediction performance across baselines and surrogates (5-fold cross-validation). Two bootstrap procedures are reported: “naïve” resamples only the CV test residuals (*B* = 1000), while the repeated-CV band spans the 10–90th percentile of the pooled score over (*B* = 20 independent 5-fold CV repeats; the same *B* repeats apply to all five models).

Model	*R* ^2^	*R*^2^ Naïve 95%	*R*^2^ Repeated CV [10–90%]	MAE (J g^−1^) [Naïve 95%]
Mean predictor	−0.025	[−0.11, +0.00]	[−0.06, −0.01]	37.19 [29.48, 44.52]
Linear (LC only)	0.327	[0.00, +0.56]	[+0.28, +0.34]	28.16 [21.67, 34.90]
Physics (η · LC · ΔH0)	0.233	[−0.25, +0.54]	[+0.21, +0.26]	29.11 [21.36, 36.35]
Random Forest (tuned)	0.326	[+0.04, +0.48]	[+0.26, +0.35]	30.18 [24.32, 37.21]
Gaussian Process (isotropic)	−0.022	[−0.32, +0.16]	[−0.11, +0.04]	37.83 [32.11, 45.19]

**Table 3:** Temporal holdout: training on records published before 2019 (*n* = 40) and testing on records published in 2019 or later (*n* = 13).

Target	Model	*R*^2^ (Test)	MAE (Test)	*n*_test_
ΔH	Linear (LC)	0.341	23.367	13
ΔH	Physics (core family)	0.199	27.399	13
ΔH	RF tuned (with year)	0.137	27.661	13
ΔH	RF tuned (no year)	0.118	27.944	13
ΔH	GP isotropic (with year)	−0.424	36.983	13
ΔH	GP isotropic (no year)	−0.453	37.105	13
log d	RF tuned (with year)	−0.571	0.297	13
log d	RF tuned (no year)	−0.294	0.286	13
log d	GP isotropic (with year)	−0.201	0.308	13
log d	GP isotropic (no year)	−0.095	0.287	13

**Table 4:** Method-level median hypervolume across (method, shell family, core family) trios. Hypervolumes were computed in the normalized objective space (each of ΔH, LC, and log *d* normalized to [0, 1] over the empirical range) with the symmetric reference point *r* = (1.5, 1.5, 1.5); the raw HV values therefore lie in [0, 1.5^3^] = [0, 3.375] rather than [0, 1]. Within each criterion, methods with identical median HVs (to three decimal places) are tied and assigned the same rank.

Rank (Nominal)	Method	Trios	HV (RF-Nominal)	HV (GP-LCB, *α* = 1)	Δ
1	Emulsion polymerization	2	2.074	1.635	−0.439
2	Suspension polymerization	1	2.042	1.069	−0.973
3	Sol–gel (silica)	2	2.029	1.673	−0.356
4	In situ polymerization	2	2.022	1.552	−0.470
5	Sol–gel (other inorganic)	1	2.019	1.237	−0.782
6	Interfacial polycondensation	2	2.018	1.406	−0.612
7	Sol–gel (titania)	1	2.014	1.176	−0.838

## Data Availability

The original contributions presented in this study are included in the article. Further inquiries can be directed to the corresponding author.

## Supplementary Materials

The following supporting information can be downloaded at https://www.mdpi.com/article/10.3390/polym18141777/s1: Table S1: Curated micro- and nano-encapsulated phase-change material database (90 records).
