# Supplementary material for: A Structure–Property Screening Framework for Polymer Shell Encapsulation of Phase-Change Materials: Random Forest and Bayesian Gaussian Process Surrogates with Multi-Objective Optimization of Polymerization Routes
Source: Polymers (Basel). 2026 Jul 21;18(14):1777. doi: 10.3390/polym18141777 (PMC13417168; doi:10.3390/polym18141777)
Supplement: Supplementary file 1 [file polymers-18-01777-s001.zip › polymers-4389573-supplementary.pdf]

Supplementary Table S1. Curated micro- and nano-encapsulated phase-change material database (90 records).

This supplementary table enumerates all 90 micro- and nano-encapsulated PCM records used in the proof-of-concept optimization framework presented in the main paper. Each record stores the encapsulation method, shell composition and class, core PCM identity and class, onset melting temperature T_m (°C), melting latent heat ΔH (J g^{-1} of capsule mass), core loading content LC (wt%), mean particle diameter d (μm), reference number, and the full citation of the primary source. Where a primary source reported a range, the arithmetic midpoint was stored (9 records, ~10 % of the dataset).

No.	Encapsulation method	Shell material	Shell class	Core PCM	Core class	T_m (°C)	ΔH (J g^{-1})	LC (wt%)	d (μm)	Ref.	Citation
1	Suspension(-like) copolymerization	Poly(styrene-co-methyl methacrylate)	Vinyl–acrylic copolymer	Paraffin wax (PRS®/Rubitherm)	Paraffin	42	88	43		19	Sanchez-Silva, L.; Rodriguez, J.F.; Romero, A.; Borreguero, A.M.; Carmona, M.; Sanchez, P. Microencapsulation of PCMs with a styrene-methyl methacrylate copolymer shell by suspension-like polymerisation. Chem. Eng. J. 2010, 157, 216–222.
2	Suspension-like polymerization	PMMA	Acrylic polymer	Caprylic acid	Fatty acid	14.3	98.7	66	4	20	Tanwar, S.; Kaur, R. Development and investigation of microencapsulated caprylic acid-based phase change materials for thermal energy storage. Int. J. Energy Res. 2021, 45, 17302–17314.
3	Suspension polymerization	TiO_2-doped PMMA	Acrylic polymer / inorganic hybrid	n-Octadecane	Paraffin (n-alkane)	25	84–142	73	10–20	21	Zhao, J.; Yang, Y.; Li, Y.; Zhao, L.; Wang, H.; Song, G.; Tang, G. Microencapsulated phase change materials with TiO2-doped PMMA shell for thermal energy storage. Sol. Energy Mater. Sol. Cells 2017, 168, 62–68.
4	Suspension-like polymerization	Poly(octadecyl methacrylate-co-methacrylic acid) (ODMA–MAA)	Acrylic copolymer	n-Octadecane (C18)	Paraffin (n-alkane)	26	93	21	1.60–1.68	22	Tang, X.; Li, W.; Zhang, X.; Shi, H. Fabrication and characterization of microencapsulated phase change material with low supercooling for thermal energy storage. Energy 2014, 68, 160–166.
5	Emulsion polymerization (UV-initiated)	PMMA	Acrylic polymer	Paraffin	Paraffin	33.6	101	61.2	0.5–2	23	Ma, S.; Song, G.; Li, W.; Fan, P.; Tang, G. UV irradiation-initiated MMA polymerization to prepare microcapsules containing phase change paraffin. Sol. Energy Mater. Sol. Cells 2010, 94, 1643–1647.
6	Mini-emulsion polymerization	PMMA	Acrylic polymer	Stearic–eicosanoic acid eutectic	Fatty-acid eutectic	57	126	69	0.046	24	Wang, Y.; Zhang, Y.; Xia, T.; Zhao, W.; Yang, W. Effects of fabricated technology on particle size distribution and thermal properties of stearic–eicosanoic acid/PMMA nanocapsules. Sol. Energy Mater. Sol. Cells 2014, 120, 481–490.
7	Photo-initiated polymerization ($FeCl_3$ initiator)	PMMA	Acrylic polymer	Stearic acid	Fatty acid	55.3	102.1	52	0.29	25	Zhang, T.; Chen, M.; Zhang, Y.; Wang, Y. Microencapsulation of stearic acid with poly(methyl methacrylate) using iron(III) chloride as photo-initiator for thermal energy storage. Chin. J. Chem. Eng. 2017, 25, 1524–1532.
8	Emulsion polymerization (RSM-optimised)	Polystyrene	Vinyl polymer	Paraffin	Paraffin	52–55	115	87.8	18	26	Sami, S.; Etesami, N. Thermal characterization of obtained microencapsulated paraffin under optimal conditions. J. Therm. Anal. Calorim. 2017, 130, 1961–1971.
9	Emulsion polymerization (RSM-optimised)	Polystyrene	Vinyl polymer	Lauric acid	Fatty acid	43.77	167.26	91.64	1.32	27	Sami, S.; Sadrameli, S.M.; Etesami, N. Thermal properties optimization of microencapsulated a renewable and non-toxic phase change material with a polystyrene shell for thermal energy storage systems. Appl. Therm. Eng. 2018, 130, 1416–1424.

No.	Encapsulation method	Shell material	Shell class	Core PCM	Core class	T_m (°C)	ΔH (J g^{-1})	LC (wt%)	d (μm)	Ref.	Citation
10	Emulsion polymerization	PMMA	Acrylic polymer	n-Docosane (C22)	Paraffin (n-alkane)	41	54.6	28		28	Alkan, C.; Sarı, A.; Karaipekli, A.; Uzun, O. Preparation, characterization, and thermal properties of microencapsulated phase change material for thermal energy storage. Sol. Energy Mater. Sol. Cells 2009, 93, 143–147.
11	In-situ / suspension polymerization	Acrylic polymer	Acrylic polymer	n-Hexadecane	Paraffin (n-alkane)					29	Su, J.F.; Wang, L.X.; Ren, L. Preparation and characterization of double-MF shell microPCMs used in building materials. J. Appl. Polym. Sci. 2005, 97, 1755–1762.
12	In-situ emulsion interfacial polycondensation	AlOOH (boehmite / aluminium oxyhydroxide)	Inorganic (metal oxyhydroxide)	Palmitic acid (PA)	Fatty acid			52–69	0.2	30	Pan, L.; Tao, Q.; Zhang, S.; Wang, S.; Zhang, J.; Wang, S.; Wang, Z.; Zhang, Z. Preparation, characterization and thermal properties of micro-encapsulated phase change materials. Sol. Energy Mater. Sol. Cells 2012, 98, 66–70.
13	Emulsion polymerization	PMMA	Acrylic polymer	n-Octacosane (C28)	Paraffin (n-alkane)	50.6	86.4	43		31	Sari, A.; Alkan, C.; Karaipekli, A. Microencapsulated n-octacosane as phase change material for thermal energy storage. Sol. Energy 2009, 83, 1757–1763.
14	Emulsion polymerization	Polystyrene	Vinyl polymer	n-Heptadecane (C17)	Paraffin (n-alkane)	21.48	136.89			32	Sari, A.; Alkan, C.; Doguscu, D.K.; Bicer, A. Micro/nano-encapsulated n-heptadecane with polystyrene shell for latent heat thermal energy storage. Sol. Energy Mater. Sol. Cells 2014, 126, 42–50.
15	Emulsion polymerization	PMMA	Acrylic polymer	Paraffin eutectic mixtures (C17–C24, C19–C18, C19–C24, C20–C24)	Paraffin eutectic	20–36	86–169			33	Sari, A.; Alkan, C.; Bicer, A. Micro/nano encapsulation of some paraffin eutectic mixtures with poly(methyl methacrylate) shell: preparation, characterization and latent heat thermal energy storage properties. Appl. Energy 2014, 136, 217–227.
16	Dispersion polymerization	Melamine-formaldehyde	Amino resin	Paraffin (n-alkane)	Paraffin		162	79	3.9	34	Khakzad, F.; Alinejad, Z.; Shirin-Abadi, A.R.; Ghasemi, M.; Mahdavian, A.R. Optimization of parameters in preparation of PCM microcapsules based on melamine-formaldehyde through dispersion polymerization. Colloid Polym. Sci. 2014, 292, 355–368.
17	In-situ polymerization	Melamine-formaldehyde (MF)	Amino resin	n-Octadecane (C18)	Paraffin (n-alkane)		120	40	4	35	Wang, X.; Zhao, T. Effects of parameters of the shell formation process on the performance of microencapsulated phase change materials. Text. Res. J. 2017, 87, 1848–1859.
18	In-situ polymerization	Melamine-formaldehyde	Amino resin	n-Tetracosane (C24)	Paraffin (n-alkane)	53	135	25	2–20	36	Naikwadi, A.T.; Samui, A.B.; Mahanwar, P.A. Melamine-formaldehyde microencapsulated n-tetracosane phase change material for solar thermal energy storage. Sol. Energy Mater. Sol. Cells 2020, 215, 110676.
19	In-situ polymerization (SMA-stabilised emulsion)	Melamine-formaldehyde	Amino resin	n-Hexadecanol (1-hexadecanol)	Fatty alcohol	51	171	79.1	10–60	37	Yin, D.; Ma, L.; Geng, W.; Zhang, B.; Zhang, Q. Microencapsulation of n-hexadecanol by in situ polymerization of melamine-formaldehyde resin in emulsion stabilized by styrene–maleic anhydride copolymer. Int. J. Energy Res. 2015, 39, 661–667.
20	In-situ polymerization	Melamine-formaldehyde	Amino resin	1-Dodecanol (lauryl alcohol)	Fatty alcohol	20	79.45	40.9	0.49	38	Kumar, G.N.; Al-Aifan, B.; Parameshwaran, R.; Ram, V.V. Facile synthesis of microencapsulated 1-dodecanol/melamine-formaldehyde phase change

No.	Encapsulation method	Shell material	Shell class	Core PCM	Core class	T_m (°C)	ΔH (J g^{-1})	LC (wt%)	d (μm)	Ref.	Citation
											material using in-situ polymerization for thermal energy storage. Colloids Surf. A 2021, 610, 125698.
21	In-situ polymerization	Poly(melamine-urea-formaldehyde) (PMUF)	Amino resin	Capric acid (decanoic acid, C10) — confirm	Fatty acid	29	61	54	0.29	39	Konuklu, Y.; Paksoy, H.O.; Unal, M.; Konuklu, S. Microencapsulation of a fatty acid with poly(melamine-urea-formaldehyde). Energy Convers. Manag. 2014, 80, 382–390.
22	In-situ polymerization	Melamine-urea-formaldehyde resin (MUF)	Amino resin	n-Dodecanol (1-dodecanol)	Fatty alcohol	19.11	135.2	68.4	38	40	Wu, N.; Xu, L.; Zhang, C. The influence of emulsifiers on preparation and properties of microcapsules of melamine-urea-formaldehyde resins with n-dodecanol as phase-change material. Adv. Polym. Technol. 2018, 37, 3492–3498.
23	In-situ polymerization	Melamine-formaldehyde	Amino resin	n-Dodecanol (bio-based, 1-dodecanol)	Fatty alcohol	28	110–181	50–83	0.71–6.28	41	Zhang, H.; Li, W.; Huang, R.; Wang, N.; Wang, J.; Zhang, X. Microstructure regulation of microencapsulated bio-based n-dodecanol as phase change materials via in situ polymerization. New J. Chem. 2017, 41, 14696–14707.
24	In-situ polymerization	Etherified melamine-formaldehyde (methanol/butanol-etherified MMF/BMF prepolymers)	Amino resin (etherified)	n-Dodecanol	Fatty alcohol	24	100	50	1–6	42	Huang, R.; Li, W.; Wang, J.; Zhang, X. Effects of oil-soluble etherified melamine-formaldehyde prepolymers on in situ microencapsulation of n-dodecanol. New J. Chem. 2017, 41, 9424–9437.
25	In-situ polymerization	Cellulose-nanofiber (CNF)-modified melamine-formaldehyde	Amino resin (CNF-modified)	1-Dodecanol	Fatty alcohol	26	100	50	1–3	43	Wu, S.; Zhang, P.; Xu, Z.; Chen, Z.; Gao, Y. Preparation of 1-dodecanol microcapsules with cellulose nanofibers-modified melamine-formaldehyde resin as a potential phase change material. Mater. Res. Express 2019, 6, 125376.
26	In-situ polymerization	Resorcinol-modified melamine-formaldehyde	Amino resin (resorcinol-modified)	n-Octadecane (C18)	Paraffin (n-alkane)				<20	44	Zhang, H.; Wang, X. Fabrication and performances of microencapsulated phase change materials based on n-octadecane core and resorcinol-modified melamine–formaldehyde shell. Colloids Surf. A 2009, 332, 129–138.
27	In-situ polymerization	Urea-melamine-formaldehyde copolymer (UMF)	Amino resin	n-Octadecane (C18)	Paraffin (n-alkane)					45	Zhang, X.X.; Tao, X.M.; Yick, K.L.; Wang, X.C. Structure and thermal stability of microencapsulated phase-change materials. Colloid Polym. Sci. 2004, 282, 330–336.
28	In-situ polymerization	Melamine-formaldehyde resin	Amino resin	n-Octadecane (C18)	Paraffin (n-alkane)					46	Su, J.F.; Wang, L.X.; Ren, L. Fabrication and thermal properties of microPCMs using melamine–formaldehyde resin as shell material. J. Appl. Polym. Sci. 2007, 105, 1816–1823.
29	In-situ polymerization (emulsion → pre-polymerization → polymerization)	Melamine-formaldehyde	Amino resin	n-Eicosane (C20)	Paraffin (n-alkane)		146.2		1.1	47	Sanchez-Silva, L.; Carmona, M.; Lopez, V.; Romero, A.; Valverde, J.L. Study of melamine-formaldehyde/phase change material microcapsules for the preparation of polymer films by extrusion. Polymers 2022, 14, 1230.
30	Emulsion-templating self-assembly + in-situ precipitation	Cuprous oxide (Cu_2O)	Inorganic (metal oxide)	n-Eicosane (C20)	Paraffin (n-alkane)					48	Gao, F.; Wang, X.; Wu, D. Design and fabrication of bifunctional microcapsules for solar thermal energy storage and solar photocatalysis. Sol. Energy Mater. Sol. Cells 2017, 168, 146–164.

No.	Encapsulation method	Shell material	Shell class	Core PCM	Core class	T_m (°C)	ΔH (J g^{-1})	LC (wt%)	d (μm)	Ref.	Citation
31	Emulsion polymerization	PMMA (poly(methyl methacrylate))	Acrylic polymer	Capric–stearic acid eutectic mixture (C-SEM)	Fatty-acid eutectic	21.37	116.25		1.3	49	Sarı, A.; Alkan, C.; Özcan, A.N. Synthesis and characterization of micro/nano capsules of PMMA/capric–stearic acid eutectic mixture for low temperature-thermal energy storage in buildings. Energy Build. 2015, 90, 106–113.
32	In-situ polymerization	Melamine-urea-formaldehyde (MUF)	Amino resin	Paraffin	Paraffin			77.1		50	Han, S.; Chen, Y.; Lyu, S.; Chen, Z.; Wang, S.; Fu, F. Effects of processing conditions on the properties of paraffin/melamine-urea-formaldehyde microcapsules prepared by in situ polymerization. Colloids Surf. A Physicochem. Eng. Asp. 2020, 585, 124046.
33	In-situ polymerization	Urea-formaldehyde (UF)	Amino resin	Paraffin	Paraffin					51	Dubey, A.K.; Dash, M.; Vasita, R.; Shukla, A.K.; Choudhary, T.; Nanda, H.S. Fabrication and thermal performance of tailored phase change microcapsules with paraffin core and urea–formaldehyde shell for thermal energy storage applications. Energy Technol. 2025, 13, e202500409.
34	In-situ polymerization (two-step: prepolymerization + polymerization)	Poly(urea-formaldehyde) (PUF)	Amino resin	Commercial paraffin (Rubitherm® RT-42)	Paraffin			90.6		52	Sánchez-Silva, L.; Lopez, V.; Cuenca, N.; Valverde, J.L. Poly(urea-formaldehyde) microcapsules containing commercial paraffin: in situ polymerization study. Colloid Polym. Sci. 2018, 296, 1449–1457.
35	In-situ polymerization (O/W emulsion + prepolymer)	Phenol-formaldehyde resin	Phenolic resin	n-Eicosane (C20)	Paraffin (n-alkane)					53	Liu, C.; Cao, H.; Jin, S.; Bao, Y.; Cheng, Q.; Rao, Z. Synthesis and characterization of microencapsulated phase change material with phenol-formaldehyde resin shell for thermal energy storage. Sol. Energy Mater. Sol. Cells 2022, 243, 111789.
36	In-situ polymerization	Poly(melamine-urea-formaldehyde) (PMUF)	Amino resin	n-Pentadecane (C15)	Paraffin (n-alkane)					54	Konuklu, Y.; Erzin, F. Preparation of pentadecane/poly(melamine-urea-formaldehyde) microcapsules for thermal energy storage applications. Int. J. Energy Res. 2019, 43, 6322–6326.
37	Interfacial polymerization (polycondensation)	Polyurea	Polyurea	n-Octadecane (C18)	Paraffin (n-alkane)			70	7.3	55	Siddhan, P.; Jassal, M.; Agrawal, A.K. Core content and stability of n-octadecane-containing polyurea microcapsules produced by interfacial polymerization. J. Appl. Polym. Sci. 2007, 106, 786–792.
38	Interfacial polymerization	Polyurea/polyurethane double-composition shell	Polyurea/polyurethane	n-Octadecane (C18)	Paraffin (n-alkane)	28.6	143		3–5	56	Lu, S.; Shen, T.; Xing, J.; Song, Q.; Xin, C. Preparation and characterization of double-composition shell microencapsulated phase change material. Colloid Polym. Sci. 2017, 295, 2061–2067.
39	Suspension/emulsion polymerization (confirm)	P(MMA-co-DVB) acrylic copolymer (confirm)	Acrylic copolymer (confirm)	Binary core (butyl stearate + paraffin)	Paraffin/ester binary	~25–50	58–87	40–60	5–15	57	Ma, Y.; Chu, X.; Tang, G.; Yao, Y. Adjusting phase change temperature of microcapsules by regulating their core compositions. Mater. Lett. 2012, 82, 39–41.
40	Interfacial polymerization	Cross-linked polyurethane	Polyurethane (cross-linked)	Butyl stearate	Fatty acid ester	22	80		10–35	58	Lu, S.; Shen, T.; Xing, J.; Song, Q.; Shao, J.; Zhang, J.; Xin, C. Preparation and characterization of cross-linked polyurethane shell microencapsulated phase change materials by interfacial polymerization. Mater. Lett. 2018, 211, 36–39.
41	Interfacial polymerization + in-situ (sol-gel for SiO_2)	Polyurea/SiO_2 & polyurethane/SiO_2 composite	Polyurea or polyurethane / SiO_2 composite	Ethyl palmitate (palmitic acid ethyl ester)	Fatty acid ester	17–22	120–122	59–61	10	59	Yin, Q.; Zhu, Z.; Li, W.; Guo, M.; Wang, Y.; Wang, J.; Zhang, X. Fabrication and performance of composite microencapsulated phase change

No.	Encapsulation method	Shell material	Shell class	Core PCM	Core class	T_m (°C)	ΔH (J g^{-1})	LC (wt%)	d (μm)	Ref.	Citation
											materials with palmitic acid ethyl ester as core. Polymers 2018, 10, 726.
42	Interfacial polycondensation (co-solvent-free; TDI + DETA)	Polyurea	Polyurea	Dodecanol dodecanoate (lauryl laurate)	Fatty acid ester	32	139	74	10–40	60	Cai, C.; Ouyang, X.; Zhou, L.; Liu, G.; Wang, Y.; Zhu, G.; Yao, J.; Militky, J.; Venkataraman, M.; Zhang, G. Co-solvent free interfacial polycondensation and properties of polyurea PCM microcapsules. Sol. Energy 2020, 199, 721–730.
43	Interfacial polymerization (HDI + chitosan, charge-attraction-assisted)	Chitosan-based polyurethane (c-PU)	Polyurethane (chitosan-based biopolymer)	Butyl stearate	Fatty acid ester	24	106		4.46	61	Gao, Y.; Geng, X.; Wang, X.; Han, N.; Zhang, X.; Li, W. Synthesis and characterization of microencapsulated phase change materials with chitosan-based polyurethane shell. Carbohydr. Polym. 2021, 273, 118629.
44	Interfacial polycondensation (IPDI + EDA)	Polyurea	Polyurea	Paraffin	Paraffin		92.5	44.5	2.42	62	Zhan, S.; Chen, S.; Chen, L.; Hou, W. Preparation and characterization of polyurea microencapsulated phase change material by interfacial polycondensation method. Powder Technol. 2016, 292, 217–222.
45	Interfacial polycondensation (TDI + DETA, aqueous SMA dispersion)	Polyurethane (PU)	Polyurethane	n-Octadecane (C18)	Paraffin (n-alkane)				5–10	63	Su, J.F.; Wang, L.X.; Ren, L.; Huang, Z.; Meng, X.W. Preparation and characterization of polyurethane microcapsules containing n-octadecane with styrene-maleic anhydride as a surfactant by interfacial polycondensation. J. Appl. Polym. Sci. 2006, 102, 4996–5006.
46	Interfacial polymerization	Polyurea/polyurethane double shell	Polyurea/polyurethane (double shell)	Binary core (butyl stearate + paraffin)	Paraffin/ester binary					64	Ma, Y.; Chu, X.; Tang, G.; Yao, Y. The effect of different soft segments on the formation and properties of binary core microencapsulated phase change materials with polyurea/polyurethane double shell. J. Colloid Interface Sci. 2013, 392, 407–414.
47	Interfacial polycondensation (polyurea system)	Polyurea	Polyurea	Butyl stearate (n-octadecanoic acid butyl ester)	Fatty acid ester				20–35	65	Liang, C.; Lingling, X.; Hongbo, S.; Zhibin, Z. Microencapsulation of butyl stearate as a phase change material by interfacial polycondensation in a polyurea system. Energy Convers. Manag. 2009, 50, 723–729.
48	Emulsion interfacial polymerization	Polyurethane (polymeric MDI + PEG)	Polyurethane (elastic)	n-Octadecane (C18)	Paraffin (n-alkane)					66	Voronin, D.V.; Sitmukhanova, E.; Mendgaziev, R.I.; Rubtsova, M.I.; Kopitsyn, D.; Cherednichenko, K.A.; Semenov, A.P.; Fakhrullin, R.; Shchukin, D.G.; Vinokurov, V. Polyurethane/n-octadecane phase-change microcapsules via emulsion interfacial polymerization. Materials 2023, 16, 6460.
49	Sol-gel process (TEOS, O/W emulsion)	Silica (SiO_2)	Inorganic (silica)	n-Octadecane (C18)	Paraffin (n-alkane)				7–16	67	Zhang, H.; Wang, X.; Wu, D. Silica encapsulation of n-octadecane via sol-gel process: a novel microencapsulated phase-change material with enhanced thermal conductivity. J. Colloid Interface Sci. 2010, 343, 246–255.
50	Sol-gel method	Silica (SiO_2, from TEOS)	Inorganic (silica)	Paraffin	Paraffin	59.26	189.24	87.5		68	Fang, G.; Chen, Z.; Li, H. Synthesis and properties of microencapsulated paraffin composites with SiO2 shell as thermal energy storage materials. Chem. Eng. J. 2010, 163, 154–159.
51	Sol-gel method (sodium silicate precursor)	Silica (SiO_2)	Inorganic (silica)	n-Octadecane (C18)	Paraffin (n-alkane)				8	69	He, F.; Wang, X.; Wu, D. New approach for sol–gel synthesis of microencapsulated n-octadecane phase

No.	Encapsulation method	Shell material	Shell class	Core PCM	Core class	T_m (°C)	ΔH (J g^{-1})	LC (wt%)	d (μm)	Ref.	Citation
											change material with silica wall using sodium silicate precursor. Energy 2014, 67, 223–233.
52	Mini-emulsion polymerization	PMMA (poly(methyl methacrylate))	Acrylic polymer	n-Eicosane (C20)	Paraffin (n-alkane)	34.66	124.7		0.135	70	Rezvanpour, M.; Hasanzadeh, M.; Azizi, D.; Rezvanpour, A.; Alizadeh, M. Synthesis and characterization of micro-nanoencapsulated n-eicosane with PMMA shell as novel phase change materials for thermal energy storage. Mater. Chem. Phys. 2018, 215, 299–304.
53	Sol-gel method	Silica (SiO_2, from TEOS)	Inorganic (silica)	Stearic acid (C18, octadecanoic acid)	Fatty acid		182.53	86.68		71	Ishak, S.; Mandal, S.; Lee, H.-S.; Singh, J.K. Microencapsulation of stearic acid with SiO2 shell as phase change material for potential energy storage. Sci. Rep. 2020, 10, 15047.
54	Sol-gel (in-situ dehydration/condensation; sodium silicate precursor)	Silica (SiO_2)	Inorganic (silica)	Paraffin wax	Paraffin		94.4	74.51		72	Zhang, Z.; Liu, Y.; Wang, J.; Sun, L.; Xie, T.; Yang, K.; Li, Z. Preparation and characterization of high efficiency microencapsulated phase change material based on paraffin wax core and SiO2 shell derived from sodium silicate precursor. Colloids Surf. A 2021, 625, 126905.
55	Sol-gel method (single-pot)	Amorphous silica (SiO_2)	Inorganic (silica)	Paraffin (nano)	Paraffin		160.86	87.83	0.19	73	Adnin, R.J.; Lee, H.S. Advancing Thermal Energy Storage: Synthesis and Thermal Performance of Silica-Encapsulated Paraffin PCMs. Molecules 2025, 30, 1698.
56	Sol-gel method	Silica (SiO_2, from MTES)	Inorganic (silica)	Myristic acid–palmitic acid (MA–PA) eutectic	Fatty-acid eutectic		169.69			74	Alva, G.; Huang, X.; Liu, L.; Fang, G. Synthesis and characterization of microencapsulated myristic acid–palmitic acid eutectic mixture as phase change material for thermal energy storage. Appl. Energy 2017, 203, 677–685.
57	Sol-gel method	Silica (SiO_2)	Inorganic (silica)	Stearic acid (C18, octadecanoic acid)	Fatty acid		169.4			75	Yuan, H.; Bai, H.; Zhang, X.; Zhang, J.; Zhang, Z.; Yang, L. Synthesis and characterization of stearic acid/silicon dioxide nanoencapsules for solar energy storage. Sol. Energy 2018, 173, 42–52.
58	Sol-gel method	Silica (SiO_2, from MTES)	Inorganic (silica)	n-Octadecane (C18)	Paraffin (n-alkane)					76	Tang, F.; Liu, L.; Alva, G.; Jia, Y.; Fang, G. Synthesis and properties of microencapsulated octadecane with silica shell as shape–stabilized thermal energy storage materials. Sol. Energy Mater. Sol. Cells 2017, 160, 1–6.
59	In-situ emulsion interfacial hydrolysis & polycondensation (TEOS)	Silica (SiO_2)	Inorganic (silica)	Paraffin	Paraffin	56.5	45.5	32.5	0.2–0.5	77	Li, B.; Liu, T.; Hu, L.; Wang, Y.; Gao, L. Fabrication and properties of microencapsulated paraffin@SiO2 phase change composite for thermal energy storage. ACS Sustain. Chem. Eng. 2013, 1, 374–380.
60	In-situ polycondensation (sol-gel; tetrabutyl titanate precursor)	Crystalline titania (TiO_2)	Inorganic (titania)	n-Eicosane (C20)	Paraffin (n-alkane)					78	Chai, L.; Wang, X.; Wu, D. Development of bifunctional microencapsulated phase change materials with crystalline titanium dioxide shell for latent-heat storage and photocatalytic effectiveness. Appl. Energy 2015, 138, 661–674.
61	Sol-gel (TEOS precursor; no acid/alkali; PVP-initiated)	Silica (SiO_2)	Inorganic (silica)	Palmitic acid (C16)	Fatty acid		109.9		0.474	79	Wang, H.; Ma, W.; Zhang, J.; Yang, Z.; Zong, D. Novel synthesis of silica coated palmitic acid nanocapsules for thermal energy storage. J. Energy Storage 2020, 30, 101402.

No.	Encapsulation method	Shell material	Shell class	Core PCM	Core class	T_m (°C)	ΔH (J g^{-1})	LC (wt%)	d (μm)	Ref.	Citation
62	Sol-gel method (TTIP precursor)	Titania (TiO_2)	Inorganic (titania)	Stearic acid (C18)	Fatty acid			64.76	0.597	80	Latibari, S.T.; Mehrali, M.; Mehrali, M.; Afifi, A.B.M.; Mahlia, T.M.I.; Akhiani, A.R.; Metselaar, H.S.C. Facile synthesis and thermal performances of stearic acid/titania core/shell nanocapsules by sol–gel method. Energy 2015, 85, 635–644.
63	Aerosol reaction + hydrothermal post-treatment	Titania (TiO_2, anatase; from TTIP)	Inorganic (titania)	n-Octadecane (C18)	Paraffin (n-alkane)		97	80		81	Fei, B.; Lu, H.; Qi, K.; Shi, H.; Liu, T.; Li, X.; Xin, J.H. Multi-functional microcapsules produced by aerosol reaction. J. Aerosol Sci. 2008, 39, 1089–1098.
64	Emulsion-templated interfacial polycondensation + ZnO impregnation	ZnO-doped brookite TiO_2	Inorganic (ZnO/TiO_2 composite)	n-Eicosane (C20)	Paraffin (n-alkane)					82	Liu, H.; Wang, X.; Wu, D.; Ji, S. Fabrication and applications of dual-responsive microencapsulated phase change material with enhanced solar energy-storage and solar photocatalytic effectiveness. Sol. Energy Mater. Sol. Cells 2019, 193, 184–197.
65	Sol-gel method	Titania (TiO_2)	Inorganic (titania)	Paraffin (n-eicosane, C20)	Paraffin (n-alkane)				0.7	83	Ghufran, M.; Huitink, D. Synthesis and thermal performance of nano-sized paraffin-based titania encapsulated PCMs via sol–gel method. J. Therm. Anal. Calorim. 2023, 148, 11629–11640.
66	Interfacial polymerization (PUA pre-shell) + liquid-phase deposition (LPD) of TiO_2	Composite titania-polyurea (TiO_2-PUA)	Inorganic-organic composite (TiO_2/polyurea)	n-Octadecane (C18)	Paraffin (n-alkane)			73		84	Zhao, A.; An, J.; Yang, J.; Yang, E.-H. Microencapsulated phase change materials with composite titania-polyurea (TiO2-PUA) shell. Appl. Energy 2018, 215, 468–478.
67	Sol-gel (in-situ polycondensation, non-aqueous emulsion templating)	Zirconium oxide / zirconia (ZrO_2)	Inorganic (zirconia)	n-Eicosane (C20)	Paraffin (n-alkane)	43.75	123.4			85	Zhang, Y.; Wang, X.; Wu, D. Design and fabrication of dual-functional microcapsules containing phase change material core and zirconium oxide shell with fluorescent characteristics. Sol. Energy Mater. Sol. Cells 2015, 133, 56–68.
68	Self-assembly method	Calcium carbonate ($CaCO_3$)	Inorganic (carbonate)	Paraffin-based binary cores	Paraffin binary	37.5		57.5	3	86	Wang, T.; Wang, S.; Luo, R.; Zhu, C.; Akiyama, T.; Zhang, Z. Microencapsulation of phase change materials with binary cores and calcium carbonate shell for thermal energy storage. Appl. Energy 2016, 171, 113–119.
69	Melt blending / impregnation (composite, not core-shell capsule)	Carbon nanotubes (CNTs) support	Carbon (CNT support matrix)	Fatty acids	Fatty acid					87	Meng, X.; Zhang, H.; Sun, L.; Xu, F.; Jiao, Q.; Zhao, Z.; Zhang, J.; Zhou, H.; Sawada, Y.; Liu, Y. Preparation and thermal properties of fatty acids/CNTs composite as shape-stabilized phase change materials. J. Therm. Anal. Calorim. 2013, 111, 377–384.
70	Interfacial polycondensation (sol-gel; TEOS, pH-controlled)	Silica (SiO_2)	Inorganic (silica)	n-Octadecane (C18)	Paraffin (n-alkane)				17	88	Zhang, H.; Sun, S.; Wang, X.; Wu, D. Fabrication of microencapsulated phase change materials based on n-octadecane core and silica shell through interfacial polycondensation. Colloids Surf. A 2011, 389, 104–117.
71	In-situ chemical deposition / sol-gel (ZnO shell)	Zinc oxide (ZnO)	Inorganic (zinc oxide)	n-Eicosane (C20)	Paraffin (n-alkane)					89	Li, F.; Wang, X.; Wu, D. Fabrication of multifunctional microcapsules containing n-eicosane core and zinc oxide shell for low-temperature energy storage, photocatalysis, and antibiosis. Energy Convers. Manag. 2015, 106, 873–885.
72	Miniemulsion polymerization	Styrene-butyl acrylate copolymer	Acrylic copolymer	n-Dodecanol	Fatty alcohol	18.4	109.2	98.4	0.1	90	Chen, C.; Chen, Z.; Zeng, X.; Fang, X.; Zhang, Z. Fabrication and characterization of nanocapsules containing n-dodecanol by miniemulsion

No.	Encapsulation method	Shell material	Shell class	Core PCM	Core class	T_m (°C)	ΔH (J g^{-1})	LC (wt%)	d (μm)	Ref.	Citation
	(interfacial redox initiation)										polymerization using interfacial redox initiation. Colloid Polym. Sci. 2012, 290, 307–314.
73	Complex coacervation	Gelatin + gum arabic	Biopolymer (protein/polysaccharide)	Coco fatty acid mixture	Fatty-acid mixture	22–34				91	Ozonur, Y.; Mazman, M.; Paksoy, H.O.; Evliya, H. Microencapsulation of coco fatty acid mixture for thermal energy storage with phase change material. Int. J. Energy Res. 2006, 30, 741–749.
74	Spray drying	Gelatin + gum arabic (acacia)	Biopolymer (protein/polysaccharide)	Paraffin wax	Paraffin		216.44			92	Hawlader, M.N.A.; Uddin, M.S.; Khin, M.M. Microencapsulated PCM thermal-energy storage system. Appl. Energy 2003, 74, 195–202.
75	Complex coacervation	Gum arabic + gelatin	Biopolymer (protein/polysaccharide)	n-Hexadecane / n-octadecane / n-nonadecane (separate cores)	Paraffin (n-alkane)		144.7			93	Onder, E.; Sarier, N.; Cimen, E. Encapsulation of phase change materials by complex coacervation to improve thermal performances of woven fabrics. Thermochim. Acta 2008, 467, 63–72.
76	Spray drying	LDPE–EVA (polyethylene–ethylene-vinyl acetate); ± carbon nanofibers	Polyolefin polymer	Rubitherm® RT27 (commercial paraffin)	Paraffin (commercial)		98.1	49		94	Borreguero, A.M.; Valverde, J.L.; Rodriguez, J.F.; Barber, A.; Cubillo, J.; Carmona, M. Synthesis and characterization of microcapsules containing Rubitherm® RT27 obtained by spray drying. Chem. Eng. J. 2011, 166, 384–390.
77	In-situ polymerization	Melamine-formaldehyde (MF) resin	Aminoplast (MF)	Paraffin wax	Paraffin	48				95	Su, W.; Darkwa, J.; Kokogiannakis, G. Development of microencapsulated phase change material for solar thermal energy storage. Appl. Therm. Eng. 2017, 112, 1205–1212.
78	Complex coacervation	Gelatin + acacia (gum arabic)	Biopolymer (protein/polysaccharide)	Paraffin	Paraffin		58			96	Hawlader, M.N.A.; Uddin, M.S.; Zhu, H.J. Encapsulated phase change materials for thermal energy storage: experiments and simulation. Int. J. Energy Res. 2002, 26, 159–171.
79	Melt coaxial electrospray	Calcium alginate (Ca-alginate)	Biopolymer (polysaccharide)	n-Nonadecane (C19)	Paraffin (n-alkane)		146.3	56	<100	97	Moghaddam, M.K.; Mortazavi, S.M.; Khayamian, T. Preparation of calcium alginate microcapsules containing n-nonadecane by a melt coaxial electrospray method. J. Electrost. 2015, 73, 56–64.
80	Coaxial electrospraying	Polycaprolactone (PCL)	Biodegradable polyester	n-Hexadecane (C16)	Paraffin (n-alkane)			96	15	98	Zhang, S.; Chen, Y.; Campagne, C.; Salaün, F. Influence of a coaxial electrospraying system on the n-hexadecane/polycaprolactone phase change microcapsules properties. Materials 2020, 13, 2205.
81	Microfluidic interfacial + free-radical polymerization	PMMA/polyurea hybrid	Acrylic polymer / polyurea hybrid	n-Hexadecane (C16)	Paraffin (n-alkane)		222.6	94.5		99	Yang, L.; Dai, L.; Ye, L.; Yang, R.; Lu, Y. Microfluidic fabrication and thermal properties of microencapsulated n-hexadecane with a hybrid polymer shell for thermal energy storage. Materials 2022, 15, 3708.
82	Emulsification–solvent evaporation	Ethyl cellulose (EC)	Biopolymer (cellulose ether)	Myristic acid	Fatty acid		122.61			100	Lin, Y.; Zhu, C.; Alva, G.; Fang, G. Microencapsulation and thermal properties of myristic acid with ethyl cellulose shell for thermal energy storage. Appl. Energy 2018, 231, 494–501.
83	Emulsifier-free sol–gel	Silica (SiO_2)	Inorganic (silica)	Capric–stearic acid eutectic	Fatty-acid eutectic	21.4	91.48			101	Song, S.; Dong, L.; Qu, Z.; Ren, J.; Xiong, C. Microencapsulated capric–stearic acid with silica shell as a novel phase change material for thermal energy storage. Appl. Therm. Eng. 2014, 70, 546–551.
84	Sol–gel	Silica (SiO_2)	Inorganic (silica)	Lauric acid	Fatty acid		165.6	85.9		102	Yuan, H.; Bai, H.; Lu, X.; Zhang, X.; Zhang, J.; Zhang, Z.; Yang, L. Size controlled lauric acid/silicon dioxide nanocapsules for thermal

No.	Encapsulation method	Shell material	Shell class	Core PCM	Core class	T_m (°C)	ΔH (J g^{-1})	LC (wt%)	d (μm)	Ref.	Citation
											energy storage. Sol. Energy Mater. Sol. Cells 2019, 191, 243–257.
85	In-situ emulsion interfacial polymerization	Poly(urea–urethane) / cellulose-nanocrystal composite	Polyurethane–urea / biopolymer composite	Methyl laurate	Fatty acid ester					103	Yoo, Y.; Martinez, C.; Youngblood, J.P. Synthesis and characterization of microencapsulated phase change materials with poly(urea–urethane) shells containing cellulose nanocrystals. ACS Appl. Mater. Interfaces 2017, 9, 31763–31776.
86	Sol–gel	Titania (TiO_2)	Inorganic (titania)	Paraffin	Paraffin					104	Cao, L.; Tang, F.; Fang, G. Synthesis and characterization of microencapsulated paraffin with titanium dioxide shell as shape-stabilized thermal energy storage materials in buildings. Energy Build. 2014, 72, 31–37.
87	Sol–gel	Titania (TiO_2)	Inorganic (titania)	Capric acid (C10)	Fatty acid					105	Nikoonahad, M.; Sadrameli, S.M.; Arabpour Roghabadi, F. Preparation and optimization of nanoencapsulated capric acid being as a renewable phase change material with TiO_2 shell as shape-stabilized thermal energy storage material. J. Therm. Anal. Calorim. 2023, 148, 10735–10747.
88	Emulsion + ionic gelation (Ca-alginate, double shell)	Double alginate (Ca-alginate)	Biopolymer (polysaccharide)	Paraffin	Paraffin					106	Németh, B.; Németh, Á.S.; Tóth, J.; Fodor-Kardos, A.; Gyenis, J.; Feczkó, T. Consolidated microcapsules with double alginate shell containing paraffin for latent heat storage. Sol. Energy Mater. Sol. Cells 2015, 143, 397–405.
89	Interfacial polymerization (W/O emulsion)	Poly(ethyl-2-cyanoacrylate)	Acrylate (cyanoacrylate) polymer	Sodium thiosulfate pentahydrate ($Na_2S_2O_3 \cdot 5H_2O$)	Inorganic salt hydrate					107	Fu, W.; Zou, T.; Liang, X.; Wang, S.; Gao, X.; Zhang, Z.; Fang, Y. Characterization and thermal performance of microencapsulated sodium thiosulfate pentahydrate as phase change material for thermal energy storage. Sol. Energy Mater. Sol. Cells 2019, 193, 149–156.
90	In-situ mineralization (self-assembly)	Calcium carbonate ($CaCO_3$)	Inorganic (carbonate)	Paraffin	Paraffin					108	Jiang, J.; Yang, W.; He, F.; Xie, C.; Fan, J.; Wu, J.; Zhang, K. Microencapsulated paraffin phase-change material with calcium carbonate shell for thermal energy storage and solar-thermal conversion. Langmuir 2018, 34, 14254–14264.

Abbreviations. Al2O3, aluminium oxide; BA, butyl acrylate; BMF, butylated melamine-formaldehyde; c-PU, crosslinked polyurethane; CaCO3, calcium carbonate; CNFs, cellulose nanofibres; EVA, ethylene vinyl acetate; Fe3O4, iron(II,III) oxide; LDPE, low-density polyethylene; MF, melamine-formaldehyde; MMF, methylated melamine-formaldehyde; MUF, melamine-urea-formaldehyde; OMA, octyl methacrylate; PCL, polycaprolactone; PMF, poly(melamine-formaldehyde); PMMA, poly(methyl methacrylate); PMUF, poly(melamine-urea-formaldehyde); PNDA, poly(N,N-diallylaniline); PU, polyurethane; PUF, poly(urea-formaldehyde); PVDF, poly(vinylidene fluoride); SiO2, silicon dioxide (silica); St, styrene; TiO2, titanium dioxide; UF, urea-formaldehyde; ZnO, zinc oxide; ZrO2, zirconium dioxide.

Notes. (i) T_m is the onset melting temperature determined by DSC (±1 °C); ΔH is the melting latent heat per unit capsule mass (J g^{-1} of capsule); LC is the core loading content expressed as a weight percentage (wt%); *d* is the mean particle or capsule diameter (μm). (ii) “ ” denotes a value not reported by the primary source. (iii) Where a primary source reported a range, the arithmetic midpoint of the interval was stored; this affects 9 records (~10 % of the dataset). (iv) Shell-class and core-class designations follow the taxonomy defined in Table 1 and Section 2.1 of the main paper. (v) Records are grouped by encapsulation method and ordered chronologically within each group.
